# Transparent Multifunctional Wearable Strain Sensor With Self‐Healing and Antibacterial Capabilities for Human Motion Detection

**DOI:** 10.1002/adhm.202503689

**Published:** 2025-10-14

**Authors:** Wenqing Chen, Wei Huang, Rohit Gupta, Abbas Heydari, Biswajoy Bagchi, Lulu Xu, Yang Xue, Eirini Velliou, Manish K Tiwari

**Affiliations:** ^1^ Nanoengineered Systems Laboratory UCL Mechanical Engineering University College London London WC1E 7JE UK; ^2^ UCL Hawkes Institute University College London London W1W 7TS UK; ^3^ Centre for 3D Models of Health and Disease Division of Surgery and Interventional Science University College London London W1W 7TY UK; ^4^ Manufacturing Futures Laboratory University College London London E20 2AE UK

**Keywords:** antibacterial, biocompatible, environment‐tolerant elastomer, self‐healing, wearable strain sensor

## Abstract

Wearable strain sensors are highly desirable due to their increasing applications in smart electronic skins and healthcare monitoring systems. Nevertheless, simultaneously integrating high stretchability, sensing linearity, stable operation under sub‐zero temperatures, and long‐term storage for conductive films remains a formidable challenge. Herein, a dual‐network polyvinyl alcohol (PVA)‐based high‐performance strain sensor that overcomes these limitations through an innovative materials design is reported. The network is constructed via synergistic cross‐linking of PVA with tannic acid (TA) and glutaraldehyde (GA), followed by the incorporation of choline acetate ionic liquid (IL) to enhance the multifunctionality of the sensor (denoted as PTGIL). The PTGIL sensor exhibits a compelling combination of properties, such as exceptional mechanical robustness (strength ≈20 MPa; elongation at break ≈900%), room‐temperature self‐healing capability, and transparency (≈88% transmittance at 550 nm). Critically, it demonstrates stable sensing performance even at sub‐zero temperatures and preserves functionality after long‐term ambient storage. The biocompatibility with human dermal fibroblasts and the antimicrobial activities against *Staphylococcus aureus* (*S. aureus*) and *Escherichia coli* (*E. coli*) confirm its safety and further support long‐term skin contact applications. Beyond conventional motion monitoring, the multifunctionality of the PTGIL sensor may help bridge soft biomechanics and healthcare applications such as rehabilitation tracking following joint ligament reconstruction and intraoperative motion‐outcome correlation analysis.

## Graphical abstract

A transparent dual‐network strain sensor integrates robust mechanical properties, room‐temperature self‐healing, and stable performance under sub‐zero conditions and prolonged storage. Its excellent biocompatibility and antimicrobial properties enable safe skin contact. Beyond conventional motion sensing, the sensor offers accurate motion tracking and holds strong potential for wearable health applications, such as joint rehabilitation monitoring in personalized healthcare systems.

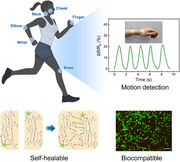

## 1 Introduction

Next‐generation wearable electronics are increasingly capable of real‐time monitoring of biochemical^[^ ^1^, ^2^ ^]^ and physical^[^ ^3^, ^4^ ^]^ signals, offering high‐resolution insights into individual health status and presenting viable alternatives to traditional clinical diagnostics. A wide range of materials, including hydrogels^[^ ^5^ ^]^ and conductive composites,^[^ ^6^ ^]^ have been developed to serve as flexible motion sensors. Among them, conductive films have garnered significant attention due to their superior biocompatibility, flexibility, and mechanical strength.^[^ ^7^ ^]^

There has been significant progress in the development of thin‐film strain sensor devices utilizing nanofillers like MXene,^[^ ^8^, ^9^ ^]^ graphene^[^ ^10^ ^]^ and carbon nanotubes.^[^ ^11^, ^12^ ^]^ However, achieving uniform dispersion of these nanomaterials within 3D polymer networks remains a substantial challenge. Poor dispersion often results in aggregate formation, which impedes charge transport during deformation cycles and leads to inconsistent gauge factors (GFs) across the working strain range.^[^ ^13^ ^]^ For instance, Yamada et al. observed two distinct GFs (0.82 for strain (ε) < 40% and 0.06 for 60% < ε < 200%) in a single‐wall carbon nanotube‐based sensor,^[^ ^14^ ^]^ while Wang et al. reported GFs of 17.5 at strain range of 0%–70% and 8962.7 at 155% strain for a carbon black‐infused thermoplastic polyurethane (TPU) sensor.^[^ ^15^ ^]^ These variations are largely attributed to differences in mechanical properties between the polymer matrix and nanofillers, which affect the reliability of deformation sensing.^[^ ^16^ ^]^ Furthermore, many nanofiller‐based sensors lack optical transparency, limiting their appeal for wearable applications where aesthetics and discreetness are critical design considerations.^[^ ^17^ ^]^

In addition to the inconsistent sensitivity, skin‐attached sensors often suffer from physical damage due to repeated use or accidental cuts. To reduce waste production and mitigate environmental pollution, self‐healing materials have attracted interest as they can restore both mechanical and electrical functionality under certain conditions, thereby extending device lifespan and reducing electronic waste. Polyvinyl alcohol (PVA) is widely used to fabricate self‐healing sensors due to its abundance of hydroxyl groups that facilitate dynamic hydrogen bonding.^[^ ^18^, ^19^ ^]^ Zheng et al. designed a hydrogel by mixing PVA, borax, silk fibroin (SF), and tannic acid (TA), with self‐healing properties via reversible borate ester bonds,^[^ ^20^ ^]^ while Kim et al. introduced a water‐activated PVA crosslinked with cellulose nanocrystal (CNC) film capable of healing through dynamic hydrogen bonding.^[^ ^21^ ^]^ However, both hydrogel‐based and film‐based sensors face environmental limitations: they gradually dry out during storage and lose function in sub‐zero temperatures due to water evaporation or freezing.^[^ ^22^ ^]^ This is important for wearable applications in colder climates. To address these limitations, ionic liquid (IL) have been proposed as additives that improve environmental stability, offering high conductivity, low vapor pressure, and excellent thermal and chemical stabilities.^[^ ^23^ ^]^ ILs can form strong hydrogen bonds with water molecules, preventing both freezing and evaporation. Yao et al. reported a lithium bromide (LiBr)‐modified strain sensor shows outstanding drying and freezing tolerances.^[^ ^24^ ^]^ Nevertheless, many IL‐based sensors are not suitable for biomedical healthcare applications due to cytotoxicity concerns.^[^ ^25^ ^]^

Herein, we introduce a dual‐crosslinked, self‐healing wearable strain sensor specifically designed to overcome limitations in current flexible electronics by enabling high‐quality signal acquisition for various human joints’ motion monitoring and ensuring long‐term wearing comfort under different environmental conditions. This advanced sensor incorporates biocompatible ionic liquid (IL) within a PVA – TA – glutaraldehyde (GA) network, representing a unique materials design strategy that combines dynamic hydrogen bonding with water retention ability to achieve superior sensing performance and multifunctionalities. The resulting PVA‐TA‐GA‐IL (PTGIL) strain sensor exhibits high sensitivity, excellent stretchability, room‐temperature self‐healing, and environmental stability, including stability at cold temperatures, drying resistance and preventing the accumulation of free radicals. These properties arise from dynamic hydrogen bonding within the crosslink network and with water molecules, and the phenolic hydroxyl groups within the polymer matrix. It also exhibits biocompatibility (>72% cell viability after 72 h treatment), antimicrobial activity (kills 90% bacteria after 2 h). Critically, this integrated approach enables safe, long‐term skin contact and positions the PTGIL sensor to potentially serve as a platform for wound‐friendly, motion‐sensitive wearable healthcare devices. By advancing the design of self‐healing materials with demonstrated biocompatibility and environmental resilience, this work offers a step forward in the development of next‐generation wearable health monitoring technologies.

## 2 Results and Discussion

### 2.1 Synthesis and Structure of the PTGIL Sensors

**Figure 1:**
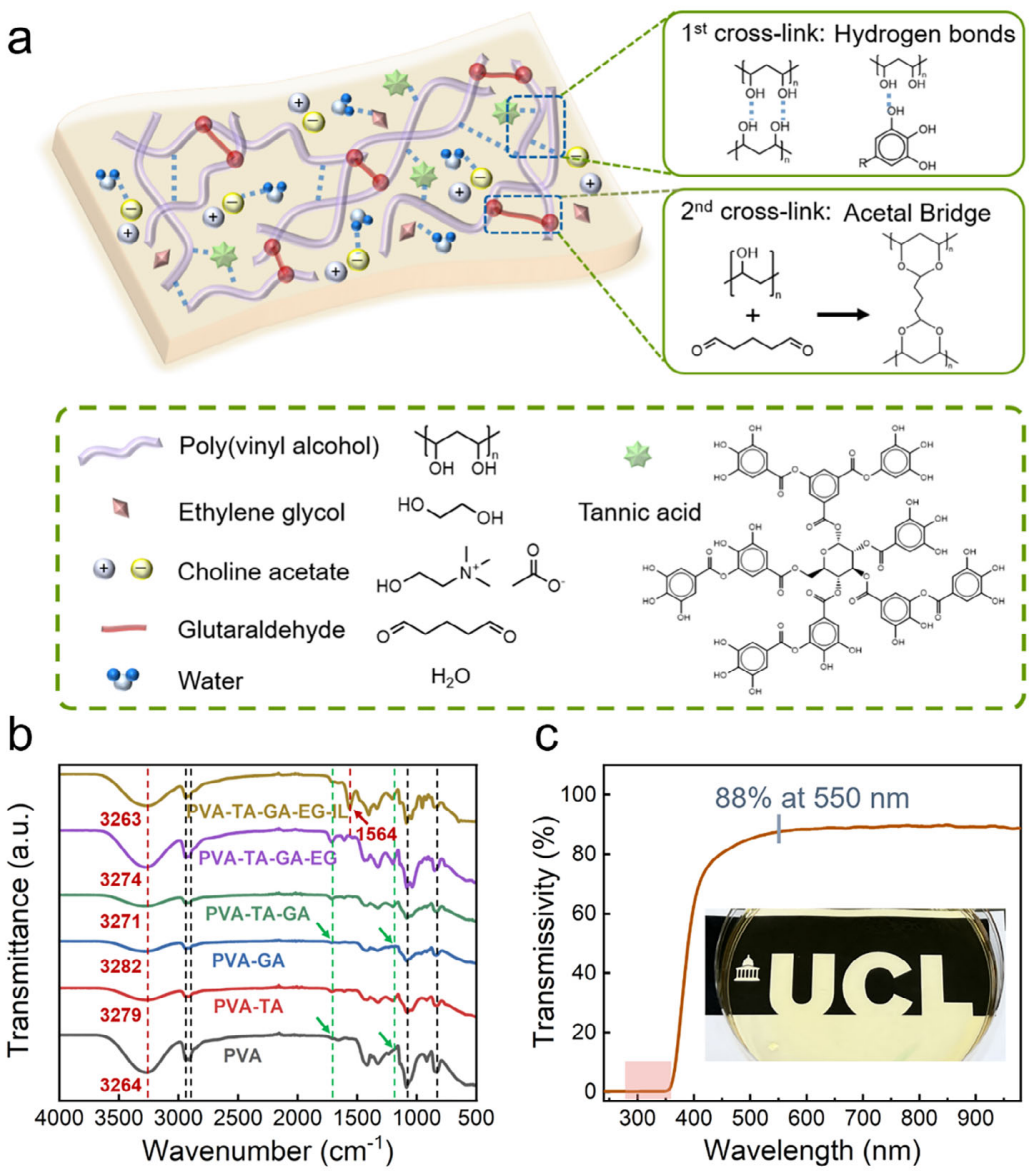
a) Schematic depicting the dual cross‐linking networks of PTGIL films. b) FTIR spectra of PVA, PVA‐TA, PVA‐GA, PVA‐TA‐GA, PVA‐TA‐GA‐EG, and PVA‐TA‐GA‐EG‐IL films. c) Optical transparency of the PTGIL strain sensors showed excellent UV absorbance over a wide wavelength range of UVA and UVB (280–360 nm, shown in red) and good transparency (88%) at the visible light spectrum (550 nm).

The PTGIL film exhibits a dual cross‐linking network, as illustrated schematically in **Figure** **1**a and Table S1 (Supporting Information), which endows the material with its novel structural and functional properties. The first network is formed through dynamic hydrogen bonding, which occurs both among hydroxyl groups within PVA chains and between PVA and TA. Complementing these interactions, choline acetate and ethylene glycol (EG) further enhance the network's cohesion by forming hydrogen bonds with water molecules embedded in the film matrix. Simultaneously, covalent acetal bridges are established through the reaction of GA with the hydroxyl groups of PVA, forming a more permanent secondary network. Notably, TA not only participates in hydrogen bonding but also modulates the pH to create an acidic environment, which facilitates efficient PVA‐GA cross‐linking while suppressing the self‐condensation of GA.^[^ ^26^ ^]^ This designed dual‐network architecture plays a pivotal role in achieving the film's mechanical robustness, flexibility, and long‐term stability, which are critical attributes for wearable sensor applications.

Fourier transform infrared spectroscopy (FTIR) was conducted to investigate molecular interactions among functional groups in a series of film formulations: PVA, PVA‐TA, PVA‐GA, PVA‐TA‐GA, PVA‐TA‐GA‐EG, and PVA‐TA‐GA‐EG‐IL. The corresponding FTIR spectra are presented in Figure 1b. The absorption peak near 839 cm^−1^ is attributed to out‐of‐plane vibrations of C─C bonds. Peaks within the 1000–1140 cm^−1^ range correspond to stretching vibrations of C‐O and C‐O‐C groups, including O‐C‐O vibrations from acetal linkages formed during PVA‐GA cross‐linking. Notably, the spectra of PVA and PVA‐GA films (highlighted in green dotted line) lack absorption peaks at 1201 and 1713 cm^−1^, confirming the absence of ester groups and C═O stretching from tannic acid. A unique peak at 1564 cm^−1^ appears only in the PVA‐TA‐GA‐EG‐IL film, indicating the presence of C‐N‐O stretching vibrations from the IL. Peaks in the 2908–2938 cm^−1^ range are assigned to C‐H stretching vibrations. Broad ─OH stretching bands are observed at 3264 cm^−1^ (PVA) and 3279 cm^−1^ (PVA‐TA). For the PVA‐GA film, the ─OH peak shifts to a higher wavenumber of 3282 cm^−1^ (red dotted line in Figure 1b), while the PVA‐TA‐GA‐EG‐IL film exhibits the lowest ─OH stretching band at 3263 cm^−1^. These shifts in vibrational frequency suggest enhanced intra‐ or intermolecular hydrogen bonding, which reduces the force constant of the chemical bonds.^[^ ^27^ ^]^ These findings indicate that hydrogen bonding between TA and PVA is weaker than the hydrogen bonds between PVA chains. Moreover, the cross‐linker GA occupies hydroxyl groups, as indicated by the decreased intensity of ─OH stretching vibrations, supporting the formation of acetal bridges during cross‐linking.^[^ ^28^ ^]^ Additional hydrogen bonds may form between the IL and water, as reported by Xue et al., who showed that choline acetate can establish strong hydrogen bonds with surrounding water molecules in lipase systems.^[^ ^29^ ^]^ These results collectively suggest that hydrogen bonding in the PTGIL film is stronger than that in the pure PVA film. All films were prepared via liquid‐phase mixing followed by solution casting.

Transparent wearable sensors designed for electronic skin (e‐skin) applications are highly desired for enhancing user comfort, aesthetics, and social acceptability. In real‐world scenarios, incorporating ultraviolet (UV) shielding into such devices is particularly important, as it not only reduces harmful UV exposure during outdoor use but also enhances the visualization of the sensor‐skin interaction. However, most existing wearable sensors lack transparent and effective UV‐filtering capabilities (Table S2, Supporting Information), which may compromise both user safety and appearance.^[^ ^19^, ^30^ ^]^ To evaluate the optical property of the PTGIL sensor, the optical transmittance was measured using a UV–vis spectrophotometer. As shown in Figure 1c (red zone), the fabricated film demonstrated near‐zero transmittance (≈0%) across the UVA and UVB spectral regions (280–360 nm), indicating UV‐shielding performance. This property is attributed to the presence of TA, which contains UV‐absorbing functional groups such as phenol, keto groups, and other chromophores.^[^ ^31^, ^32^ ^]^ Additionally, the PTGIL film exhibits high transparency (88%) across the visible light spectrum (550 nm), aligning well with the visual and aesthetic requirements for wearable motion detection technologies.

### 2.2 Mechanical Properties and Electrical Conductivities

**Figure 2:**
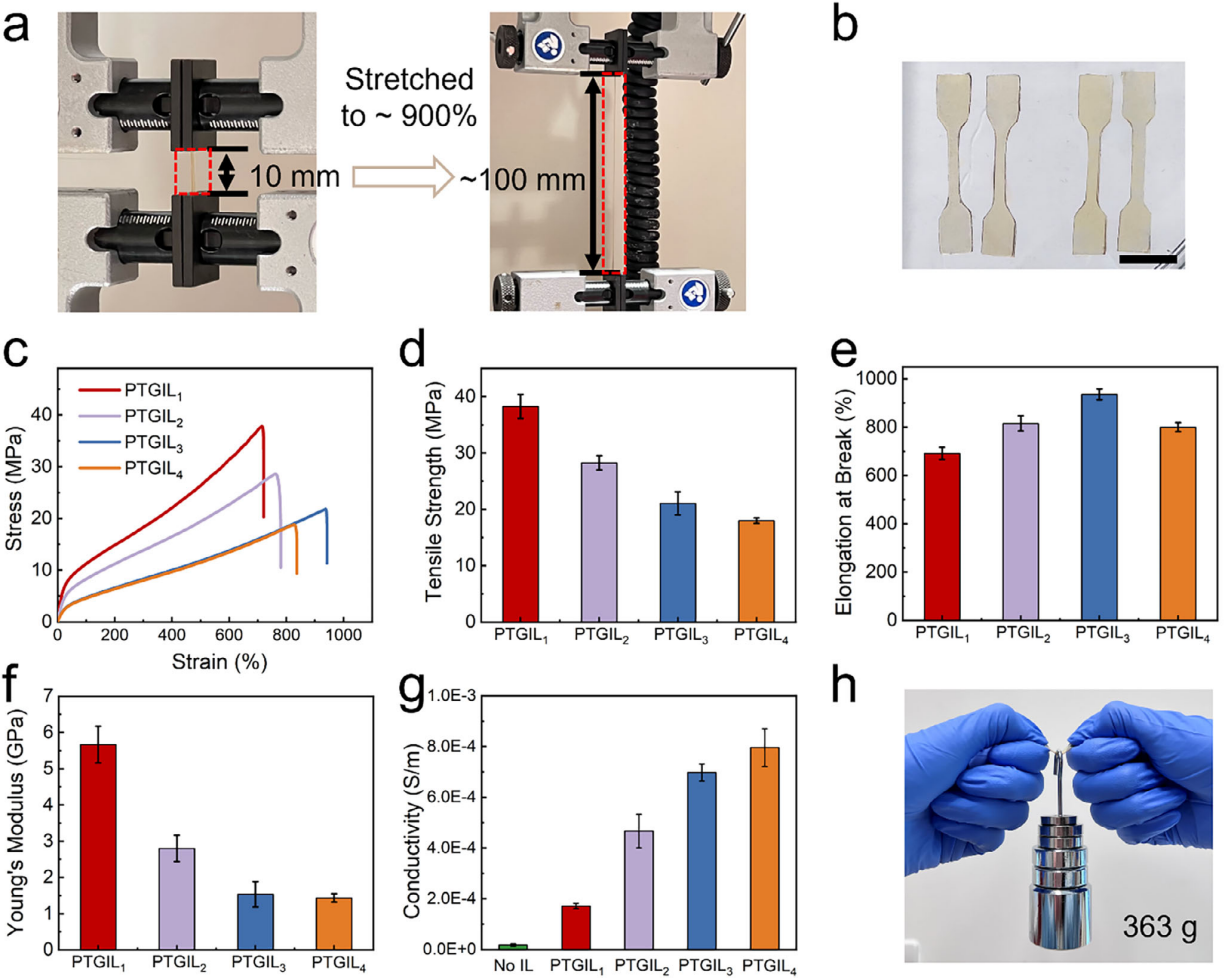
Mechanical characterization and electrical conductivity of the PTGIL films. a) Photographic images of PTGIL (e.g., PTGIL_3_) film before and after stretching up to 900% in Instron machine. b) Photographic images of dumbbell specimens (scale bar: 10 mm). c) Tensile stress‐strain curve of different IL concentrations of PTGIL samples. d) Ultimate tensile strength, e) Elongation at break, and f) Elastic modulus of the samples with different IL loadings. g) Effect of the IL concentration on the electrical conductivity of the films. h) Photographic images of a 363‐g weight‐lift demonstration using PTGIL_3_ film. Error bars represent the standard deviation (SD) of the mean for *n* = 3 replicates.

Wearable sensors designed for dynamic body regions, such as the ankle, wrist, knee, and fingers, must exhibit a combination of softness, flexibility, and robust mechanical strength to accommodate frequent bending and motion. To evaluate the mechanical performance of the PTGIL films, tensile tests were conducted at room temperature using a stretching speed of 20 mm min^−1^ in accordance with the JIS K 6251 standard for thermoplastic materials (**Figure** **2**a,b). The resulting stress‐strain curves are presented in Figure 2c; all samples demonstrated remarkable stretchability (>690% elongation at break) and high tensile strength (>15 MPa). It is expected that the presence of the hydrogen bonding and the cross‐linked PVA‐GA polymer network forms the structural backbone of the films. As shown in Figure 2d, a clear inverse relationship was observed between IL content and mechanical strength: increasing the IL concentration (from PTGIL_1_ to PTGIL_4_, see Table S1, Supporting Information) led to decreased polymer network density, thereby reducing mechanical tensile strength (from 38 to 17 MPa).^[^ ^33^ ^]^ The Young's modulus for these samples is calculated from the initial slope of the stress‐strain curve and shown in Figure 2f. The results in Figure 2g highlight the influence of IL concentration on the conductivity of the films. Conductivity increased with rising IL content, particularly up to PTGIL_3_. Beyond this point, the rate of improvement slowed, with PTGIL_4_ achieving a maximum conductivity of ≈0.0008 S m^−1^. Notably, PTGIL_3_ represented the most effective concentration, as the incremental gains between PTGIL_3_ and PTGIL_4_ were far smaller than the substantial improvements observed between the lower concentrations (i.e., between the IL‐free sample and PTGIL_1_, PTGIL_1_ and PTGIL_2_, and PTGIL_2_ and PTGIL_3_). Notably, there is huge gap between the conductivities of IL‐free samples and PTGIL_1_ samples, indicating that IL is the source of conductivity of the sensors.

Among the tested formulations, PTGIL_1_ demonstrated the highest tensile strength. However, its low IL content significantly reduced its stretchability and electrical conductivity, limiting its suitability for sensing applications that depend on efficient signal transduction. By contrast, PTGIL_3_ achieved an optimal balance of properties, maintaining robust mechanical performance with a tensile strength of 20 MPa while exhibiting excellent stretchability (≈900%, Figure 2e). These values significantly surpass those of existing wearable strain sensors, which typically exhibit tensile strengths in the kilopascal range.^[^ ^18^, ^34^ ^]^ Additionally, its higher IL concentration enhanced electrical conductivity (compared to films loaded with less IL), making it better suited for applications requiring reliable and sensitive movement detection. PTGIL_3_ was therefore selected as the representative formulation for subsequent analysis based on its balanced combination of mechanical performance, conductivity, and material stability.

Moreover, PTGIL_3_ demonstrated the consistent mechanical behavior underwent a 363‐g weight‐lift demonstration and consecutive loading‐unloading cycles at 30% and 100% strain (Figure 2h; Figure S3, Supporting Information), demonstrating the robust fatigue resistance and structural integrity. The results show that the stretched film can be adequately restored to its original state through energy dissipation. While there is a noticeable decrease in stress and an increase in plastic deformation during the first cycle, the hysteresis curve diminishes significantly after the initial loading/unloading (as shown in Figure S3, Supporting Information) and then stabilizes in subsequent cycles. The substantial hysteresis loop observed during the initial loading‐unloading cycle is primarily attributed to molecular chain rearrangement and the reformation of dynamic bonds within the film.^[^ ^35^, ^36^ ^]^ Once these bonds rearrange under the initial strain, the film's network structure is not further damaged under repeated strong tensile forces. This leads to effective energy dissipation with low hysteresis in later cycles, demonstrating the film's capacity for stable cyclic sensing performance. Overall, this trade‐off between mechanical flexibility and functional conductivity made PTGIL_3_ (referred to as PTGIL hereafter) the suitable concentration for integration into wearable strain sensors designed for real‐time motion detection.

### 2.3 Sensing Performance and Environmental Tolerance

**Figure 3:**
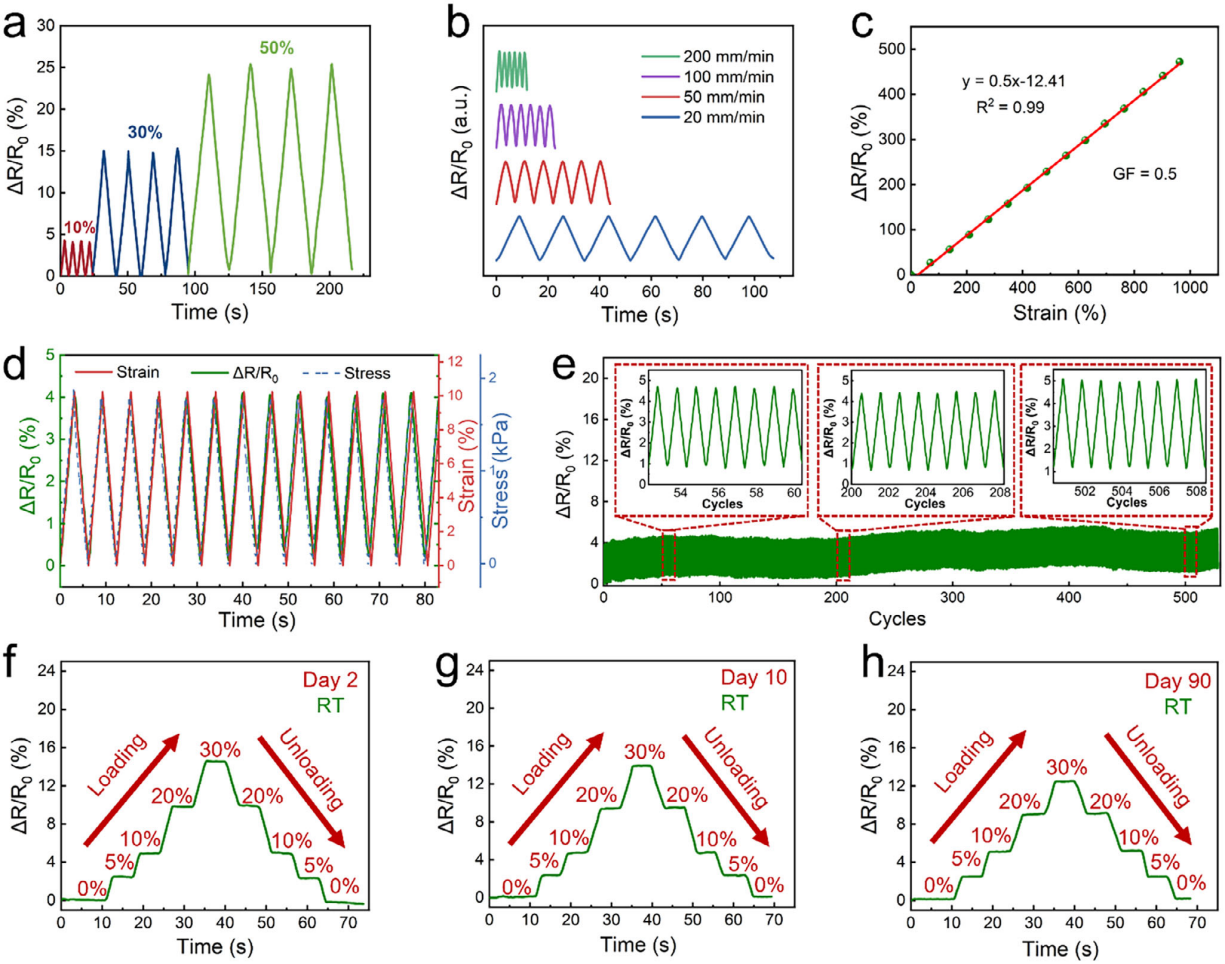
a) Dynamic electrical responses of the PTGIL strain sensor under the strain of 10%, 30%, and 50%. b) Dynamic electrical responses under different testing speeds from 20–200 mm min^−1^ at a strain of 30%. c) Relative resistance change (Δ*R*/*R*_0_) over a strain of 0%–950% and the corresponding GF of the strain sensor (Green points are the tested signal, while the red line is the trend line generated by Origin). d) The consistency of Δ*R*/*R*_0_ (green line), corresponding strain (red line), and stress (blue line) at a strain of 10%. e) The Δ*R*/*R*_0_ of the strain sensor, under 10% for ≈500 cycles at the speed of 20 mm min^−1^. (Average signal drift = 1.14%). f–h) The Δ*R*/*R*_0_ of PTGIL sensor upon responding to the stepwise strain (0%–30%) at the speed of 20 mm min^−1^ used f) sensors made after 2 days tested at room temperature, g) sensors made after 10 days tested at room temperature, h) sensors made after 90 days tested at room temperature.

The sensing capabilities of PTGIL wearable strain sensors were evaluated by subjecting them to multiple stretching and releasing cycles using an Instron tensile machine. Various stretching and releasing cycles were then applied on the PTGIL sensors, varying in strain (ranging from 10% to 50%) and at a stretching speed of 20–200 mm min^−1^, as shown in **Figure** **3**a,b. Real‐time resistance data were recorded using a multimeter, revealing stable and repeatable relative resistance changes (Δ*R*/*R*_0_) throughout testing. The working mechanism is attributed to the geometrical effect of the PTGIL sensors, resulting in a strain‐induced modulation of ion transport pathways within the film. When stretched, the film experiences an increase in conduction path length and a reduction in cross‐sectional area, leading to a measurable rise in resistance due to hindered ion mobility.^[^ ^16^ ^]^ A limit of detection study was shown in Figure S4 (Supporting Information), suggesting that the lowest amount of strain which is statistically distinguishable from the zero‐level is 0.26%. The sensitivity of the PTGIL sensor was investigated by plotting relative resistance change against applied strain, demonstrating a linear response within the strain range of 0%–900% with a GF of 0.5 (Figure 3c). The linearity (*R* ^2^ = 0.99) offers an advantage for wearable strain sensors, enabling intuitive and accurate strain quantification. This linearity is attributed to the relatively low hysteresis and uniformly narrowed conductive pathway. The PTGIL sensor demonstrates a consistent response to corresponding strain and stress throughout several stretching and releasing cycles, within the 0%–10% tensile strain range, as shown in Figure 3d. It was observed that the resistance of the sensor could almost be recovered after release, indicating its acceptable hysteresis and linearity.

To assess long‐term durability, the sensor was subjected to more than 500 consecutive loading‐unloading cycles at 10% strain. The electrical output was shown in Figure 3e, confirming mechanical resilience and signal integrity. However, it can be noted that the resistance change signals of the sensor were not completely stable over the 500 cycles, with an average drift of 1.14%. This is a common phenomenon in polymer‐based sensors. There are three main reasons of causing the drift for a elastomer: first, the inherent viscoelasticity of elastomers could lead to hysteresis, therefore causing signal baseline drift;^[^ ^37^ ^]^ second, the speed of stretching and relaxation also affects the distribution of residual stresses within the elastomer, and the hysteresis would be exacerbated in highspeed strain scenarios;^[^ ^38^ ^]^ third, the lack of fixtures and highly stable adhesive of the interfaces between the electrodes and the elastomers represent a potential factor for affecting baseline stability.^[^ ^39^ ^]^ To use this sensor for longer period of time, we can adopt for drift correction that would ensure proper motion monitoring. Further dynamic evaluation under stepwise strain loading (0%–30%, 5 s hold per level) demonstrated minimal signal drift during cycling (Figure 3f). The overall sensing response remained highly stable and reliable.

Furthermore, the drying resistance property and freezing tolerance could significantly impact the extension of sensor service life. Unlike conventional hydrogels and soft films, the PTGIL sensor retained functionality after 10, and 90 days of storage (ambient laboratory conditions), with no significant signal degradation during stretch sensing testing (Figure 3g,h). Notably, its anti‐drying performance surpasses many previously reported sensors. For example, Yao et al. described a hydrogel strain sensor that slightly increased in mass after 48 h of exposure to ambient air (25 °C, 58% RH), yet PTGIL maintains both mass and sensing performance over significantly longer durations.^[^ ^24^ ^]^ Beyond high sensing performance at room temperature, the PTGIL sensor exhibits outstanding environmental stability, maintaining performance under sub‐zero temperatures, as confirmed through stepwise tensile testing in a liquid nitrogen‐cooled chamber (Instron 3119–600). As shown in Figure S5 (Supporting Information), each sensor demonstrates repeatable output signals throughout the loading and unloading cycle, indicating the long‐term stability of the PTGIL sensor in an open environment and wide working temperature spectrum. These environmental advantages are credited to the incorporation of EG and IL, which act as low‐volatile cryoprotectants. These agents replace water molecules, form robust hydrogen bonds, and preserve ion transport pathways, thereby enhancing water retention, freeze tolerance, and operational lifespan.^[^ ^40^, ^41^ ^]^ To further assess environmental stability, sensors stored for 60 days were evaluated at −20 °C. These samples retained the ability to detect strain and nearly returned to baseline after release; however, their performance was reduced and less stable compared to freshly prepared samples (Figure S6, Supporting Information). These results indicate that while the sensors exhibit promising long‐term usability, achieving simultaneous stability during extended storage and under freezing conditions remains a challenge for future optimization.

Excessive accumulation of free radicals and associated oxidative stress also represent critical environmental challenges for strain sensors. These factors can significantly compromise device performance by inducing undesirable changes such as increased rigidity or surface tackiness, ultimately reducing mechanical flexibility and impairing electrical functionality. Moreover, prolonged exposure to free radicals poses risks to surrounding skin tissues. Therefore, addressing oxidative degradation is essential for ensuring the long‐term reliability and durability of wearable strain sensing technologies. Incorporating antioxidant functionality into stretchable sensors offers a promising strategy to mitigate these effects by scavenging reactive oxygen species and enhancing material longevity.^[^ ^42^ ^]^ To evaluate the antioxidant capacity of the PTGIL films, a 2,2‐Diphenyl‐1‐picrylhydrazyl (DPPH) radical scavenging assay was conducted (Figure S7, Supporting Information). The results showed that with increasing concentrations of the film extract, the radical scavenging activity rose significantly, reaching ≈89% after 30 min of incubation. This pronounced antioxidant behavior is attributed to the phenolic hydroxyl groups present in TA, which act as effective free radical terminators by reducing purple DPPH radicals to yellow diphenyl‐picrylhydrazine.^[^ ^43^ ^]^ These findings confirm the excellent anti‐oxidative properties of the PTGIL sensors, supporting their suitability for long‐term, skin‐interfaced wearable applications.

Together, these results demonstrate that the PTGIL sensor combines high sensitivity, long‐term durability, and environmental robustness, which makes it a compelling candidate for next‐generation wearable electronics and long‐term physiological monitoring in real‐world conditions.

### 2.4 Motion Detection

**Figure 4:**
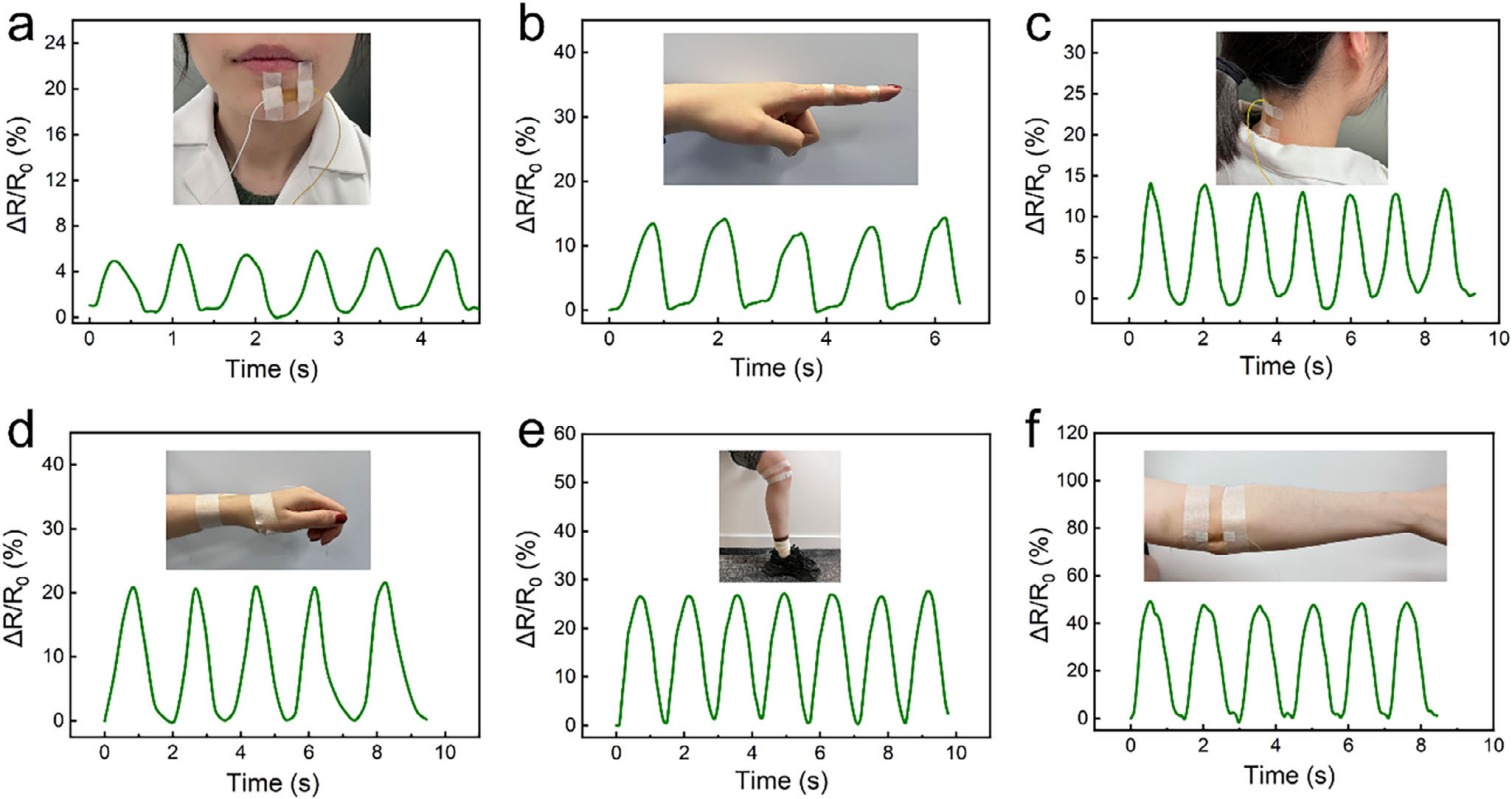
The performance of PTGIL sensors in monitoring human motions. Relative resistance change (Δ*R*/*R*_0_) in response to a) smiling, b) finger bending, c) nodding, d) wrist bending, e) knee bending, and f) elbow bending.

To evaluate the performance of the flexible and transparent PTGIL strain sensor in monitoring human motion, the sensor (PTGIL_3_, see Table S1, Supporting Information) was affixed to the skin using surgical tape and connected to conductive wires, as illustrated in **Figure** **4**; Figures S8 and S9 (Supporting Information). The device effectively captured strain signals associated with various joint movements and facial expressions, highlighting its sensitivity to both subtle and large‐scale deformations. As shown in Figure 4a, the sensor successfully detected facial muscle contractions and relaxations, such as those associated with smiling, indicating its ability to monitor fine‐scale strain variations. Similarly, the sensor demonstrated high responsiveness to finger bending (Figure 4b) and was able to distinguish head‐nodding movements (Figure 4c), underscoring its capability to track dynamic, localized motion. Further experiments involved sensor placement on the wrist, knee, and elbow to assess the detection of larger‐scale limb movements. Notably, the magnitude of resistance change increased with greater bending angles. For instance, wrist bending (≈60°) produced a modest resistance shift (Figure 4d), while knee bending (≈90°) resulted in a resistance increase of up to 30% (Figure 4e). Elbow motion produced even greater changes in resistance (Figure 4f), demonstrating the sensor's ability to differentiate bending degrees. These variations in resistance arise from geometric deformation during stretching, which reduces the cross‐sectional area of the film and restricts ion mobility, thus increasing electrical resistance. Overall, the PTGIL sensor exhibited reproducible and distinct signal patterns across multiple body movements, confirming its potential for applications in wearable electronics and health monitoring technologies.

### 2.5 Self‐Healing Properties

Wearable smart sensors are revolutionizing healthcare and motion‐tracking technologies by enabling real‐time monitoring of human movement and joint dynamics. However, their durability remains a critical limitation. Many current sensors are prone to damage from routine wear, mechanical stress, or accidental cuts issues that can compromise performance or lead to device failure.^[^ ^24^, ^35^ ^]^ To overcome these challenges, the integration of self‐healing materials offers a compelling solution. Inspired by the natural regenerative capabilities of human skin, self‐healing sensors can autonomously repair damage, restore both mechanical and electrical functions, and extend operational lifespan without external intervention. Previous studies have reported various self‐healing hydrogel‐based strain sensors; however, many require elevated temperatures to initiate healing, limiting their practical usability in real‐world wearable applications (Table S2, Supporting Information). For instance, Huang et al. developed a PVA‐based ionic organohydrogel strain sensor that required heating to 40 °C to enable the materials‘ self‐repair.^[^ ^44^ ^]^ Similarly, Dang and colleagues fabricated a flexible strain sensor based on carboxymethyl cellulose (CMC) that exhibited excellent self‐healing properties, but required heating to 80 °C to re‐establish its internal network.^[^ ^45^ ^]^ Applying heat directly to wearable sensors during use is impractical and inconvenient, often necessitating additional steps or external devices, which may affect user comfort and limit on‐demand repair.

**Figure 5:**
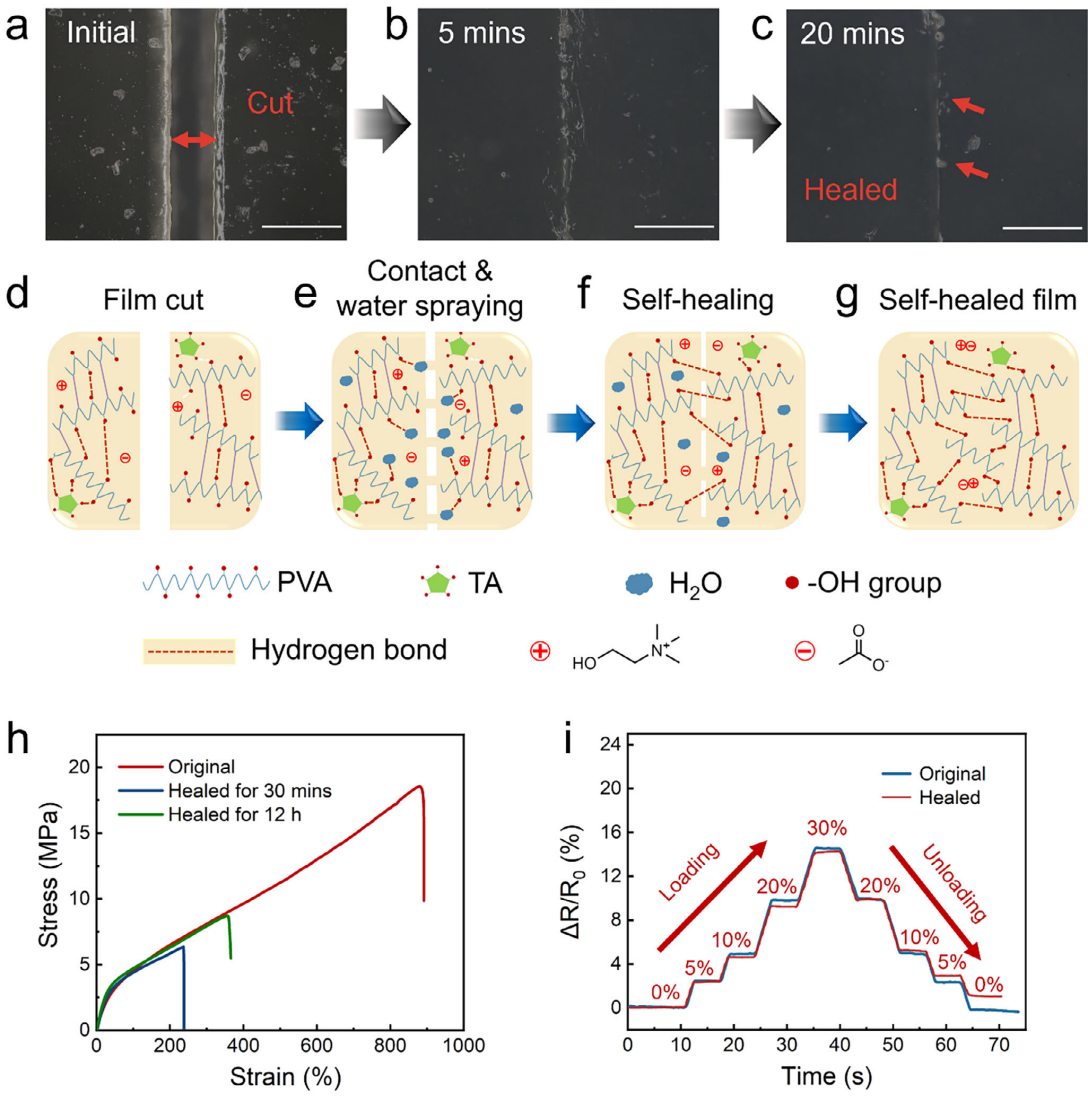
3D microscope images of the PTGIL sensor water‐induced self‐healing: a) Cut into two pieces. b) Self‐healing after 5 min. c) Self‐healing after 20 min. (Scale bar: 200 µm). d–g) Schematic illustration of the water‐induced self‐healing mechanism at room temperature. h) Tensile test of the water‐induced self‐healing film at room temperature, compared to the original film. i) Relative resistance change (Δ*R*/*R*_0_) of the original sensor and healed sensor (healed for 30 min) in responding to stepwise stain from 0%–30%.

In this study, we demonstrate the room‐temperature self‐healing capabilities of the PTGIL strain sensor. As illustrated in **Figure** **5**, the sensor was intentionally severed using a razor blade (see Figure 5a,d) and subsequently rejoined with a small application of deionized water (Figure 5b,e). Upon hydration, the cut surfaces swelled and transitioned into a hydrogel state, enabling the mobility of PVA chains and the reformation of dynamic hydrogen bonds. After 20 min at room temperature, the damaged interface nearly healed (Figure 5c,f,g). The observed FTIR shifts in the 3000–3700 cm^−1^ region reflect the dynamic hydrogen bonding interactions driving the self‐healing process (Figure S10, Supporting Information). During the initial phase (0–10 min), chain diffusion and interfacial contact promote formation of strong, dense hydrogen‐bonded clusters within the sensor,^[^ ^46^, ^47^ ^]^ resulting in a red shift (i.e., 3263 to 3250 cm^−1^). Physically, the film with 10 min of healing time is not as robust as the original film. With longer healing times (10–30 min), these dynamic hydrogen interactions reorganize into a more equilibrated and spatially distributed network, allowing the film to gradually recover toward its original state and leading to a blue shift (i.e., 3250 to 3266 cm^−1^). Electrostatic interactions between anions and cations may further contribute to the healing process.^[^ ^48^ ^]^ The spectral evolution aligns with the physical healing time in the range of 20–30 min, confirming the dynamically reversible interactions are central to the self‐healing mechanism of the PTGIL strain sensor.

To quantify healing performance, mechanical and electrical evaluations were conducted. The film recovered over 35% of its tensile strength after 30 min, with a maximum healing efficiency approaching 50% (Figure 5h). Notably, the healed sensor retained excellent flexibility; demonstrating bending, stretching, and twisting capabilities comparable to its pristine counterpart (Figure S11, Supporting Information). Electrical measurements further confirmed robust functionality, with the sensor maintaining stable signal output under 30% strain and achieving 97% electrical self‐healing efficiency (Figure 5i). These findings highlight the PTGIL sensor's potential to meet the demanding requirements of durable, self‐healing wearable electronics.

### 2.6 Biocompatibility and Antibacterial Study

The biocompatibility of wearable electronic devices is crucial for personalized healthcare applications. However, many existing wearable strain sensors incorporate toxic components, such as LiBr‐based sensors,^[^ ^24^ ^]^ that can compromise user comfort and are unsuitable for long‐term use. To evaluate the safety of the PTGIL strain sensor, which is designed for direct skin contact, a cytotoxicity study was conducted using human dermal fibroblasts (HDF).

**Figure 6:**
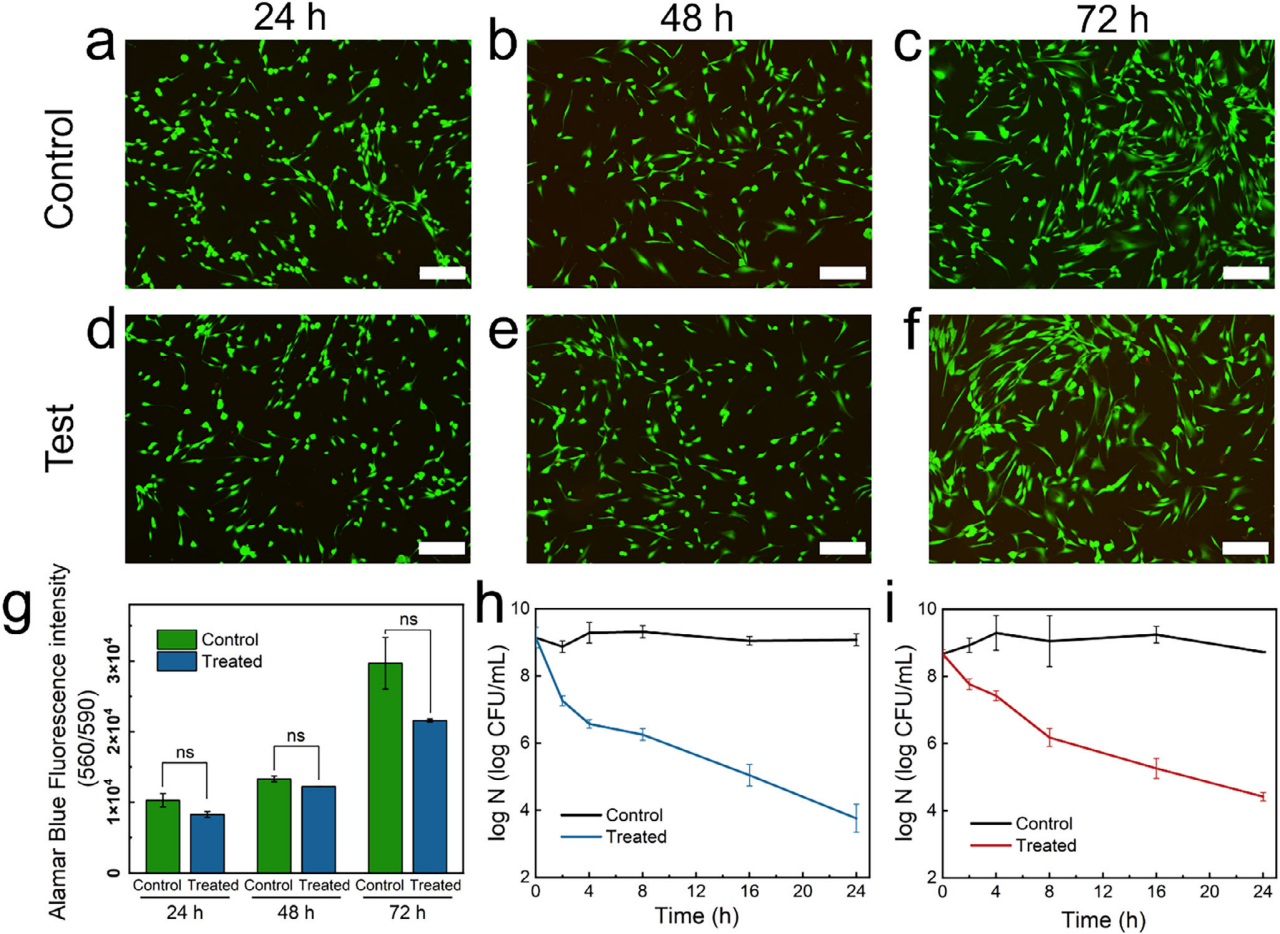
a–c) Fluorescence images after 24, 48, and 72 h incubation of untreated HDF cells. d–f) Fluorescence images of HDF cells, which were treated with PTGIL film extract for 24, 48, and 72 h. (Scale bar: 500 µm). g) Alamar Blue fluorescence intensity of the HDF cells line treated with the sensor's extract for 24, 48, and 72 h. Two‐way ANOVA coupled with Tukey's test was conducted to determine statistically significant differences (*p* < 0.05) between control and treated samples at different days. Inactivation kinetics of h) *S. aureus* and i) *E.coli* treated with film for 24 h. Error bars represent the standard deviation (SD) of the mean for *n* = 3 replicates.

In **Figure** **6**, the cell proliferation of HDF cells cultured in pure Dulbecco's Modified Eagle Medium (DMEM) media and exposed to 24, 48, and 72 h film extractions was illustrated. Live cells, identified by green‐fluorescent calcein acetoxymethyl ester (calcein‐AM) indicating their intracellular esterase activity, and dead cells, highlighted in red‐fluorescent ethidium homodimer‐1 signaling plasma membrane integrity loss, are depicted.^[^ ^49^ ^]^ As expected, HDF cells were able to grow with both DMEM media and sensor extracts (Figure 6). Comparative analysis in fluorescent images (Figure 6a–f) suggests no discernible variance in cell morphology or confluence among the treated groups and the control, suggesting the biological safety of the PTGIL sensor for healthcare applications. Additionally, the cell viability of the sensor extracts was also investigated using the Alamar Blue assay. Figure 6g indicates no significant difference in fluorescence intensity between the control and treated groups among all three time scales (24, 48, and 72 h). Notably, the live‐cell percentage of human dermal fibroblast cells surpassed 72% following treatment with 72 h extracts. Furthermore, the continual cell growth observed during treatment with sensor extracts from 24 to 72 h confirms the minimal cytotoxicity and excellent biocompatibility of the PTGIL sensor.

This flexible wearable sensor is designed to contact human skin and help with postoperative rehabilitation of human joints. Therefore, wearable sensors with the potential to protect operational wounds from bacterial infection would be more attractive. The antibacterial effect of the PTGIL sensor was assessed through 24 h bacterial inhibition test using both *Staphylococcus aureus* (*S. aureus*, gram‐positive bacteria) and *Escherichia coli* (*E. coli*, gram‐negative bacteria). The PTGIL film was directly immersed in bacterial suspensions, and the number of live bacteria at each time point was obtained by using the colony forming unit (CFU) method. Figure 6h,i represent the inactivation kinetics of *S. aureus* and *E. coli* during 24 h, and it could be observed that the PTGIL sensor exhibits an outstanding antibacterial property. Notably, after incubation for 2 h, the survival of *S. aureus* and *E. coli* exhibited a 1.6 log and 1.1 log decrease respectively, which indicates a high efficiency in killing more than 90% of bacteria within 2 h. Gradual bacterial inactivation were witnessed on both *S. aureus* and *E.coli* during further incubation. At the end of 24 h incubation, there was a significant population reduction of *S. aureus* (>5 log CFU mL^−1^), while the inhibition for *E. coli* was 4.3 log CFU mL^−1^. These huge decreases demonstrate PTGIL sensor could kill more than 99.99% of both gram‐positive and gram‐negative bacteria within 24 h. Additionally, these results suggest that *S. aureus* exhibited higher susceptibility than *E.coli*, potentially attributed to the complex structure of the cell walls of *E. coli*. The cell wall of *S. aureus* consists entirely of peptide polyglycogen. In contrast, the cell wall of *E. coli* comprises a thin layer of peptide polyglycogen and an outer lipopolysaccharide layer. This outer lipopolysaccharide layer could act as a potential barrier against foreign molecules.^[^ ^50^ ^]^ The remarkable antibacterial properties of PTGIL films stem from combining the antimicrobial effects of choline acetate and TA. The antimicrobial activity of TA encompasses various mechanisms, including enhanced membrane permeability, destabilization of the cytoplasmic membrane, and enzyme inhibition through interaction with bacterial proteins.^[^ ^51^ ^]^ Owing to the interaction with the ionic species, the membrane of bacterial cells is disrupted by choline acetate.^[^ ^52^ ^]^ These multiple modes of antibacterial actions contribute to a reduction in antimicrobial resistance. Consequently, the PTGIL strain sensor exhibits enhanced security and reliability in wearable electronics applications.

## 3 Conclusion

Overall, we have developed a wearable strain sensor that is transparent, highly stretchable, biocompatible, resilient to environmental changes, and self‐healable at room‐temperature. Engineered from a dual cross‐linked PVA composite and fabricated through a simple one‐pot fabrication method, the sensor integrates a suite of multifunctional properties essential for pertinent wearable sensing applications. The sensor exhibits high optical transparency (>88% in the visible range), excellent stretchability (≈900%), robust mechanical strength (20 MPa), and maintains reliable sensitivity after 90 days of storage. Notably, it functions stably at sub‐zero temperatures (−20 °C), resists dehydration, offers strong antioxidant and antimicrobial properties, and is inherently biocompatible; ensuring not only functional stability but also safety for direct skin contact.

These attributes position the sensor as an ideal interface between human motion and digital health systems. It can be seamlessly worn on joints such as fingers, enabling precise motion tracking during rehabilitation after ligament injuries. By delivering real‐time, high‐fidelity movement data, the sensor could provide clinicians with valuable insight into joint flexibility. Altogether, this work represents a potential step toward intelligent, skin‐conformal electronics that empower personalized, responsive healthcare through material innovation. Further work is needed to enhance sensitivity, reducing hysteresis, and improving storage robustness of these films for applications in intelligent rehabilitation and multimodal human–machine interaction. These efforts will provide both theoretical and practical foundations for developing high‐performance, biocompatible, and sustainable smart electronic systems.

## 4 Experimental Section

### Materials

PVA (Mw 89000‐98000), TA, EG, choline acetate, GA (50 wt.%), fetal bovine serum (FBS), and plate count agar (NutriSelect Plus) were all purchased from Sigma–Aldrich. Advanced DMEM, AlamarBlue Cell Viability Reagent, AlamarBlue Cell Viability Reagent, BD Difco dehydrated culture media: Columbia Broth, GlutaMAX (200 mM 100X), LIVE/DEAD viability/cytotoxicity kit (L3224), and phosphate‐buffered saline (PBS) tablets were all purchased from Thermofisher Scientific. All the chemicals were used as received.

### Sensor Fabrication Process

The wearable strain sensor was prepared using a straightforward two‐step solution casting and solvent evaporation (see Figure S1 and Table S1, Supporting Information). To start with the sensor fabrication, aqueous solutions were prepared by dissolving PVA in distilled water overnight with a concentration of 10 wt.%. Next, 1 wt.% tannic acid was added to the PVA solution and stirred at 90 °C for 3 h to obtain a homogenous solution. Then, an appropriate amount of ethylene glycol, choline acetate and cross‐linker glutaraldehyde were added to the PVA‐TA solution and continually stirred at 70 °C for 2 h. Subsequently, the mixture was cast in a glass mould and kept at room temperature overnight.

### FTIR and UV–vis Spectroscopy

FTIR was performed using an FTIR spectrometer (iS50 FT‐IR, NicoLet). The optical transmittance spectra of PTGIL films were recorded by using an Orion AquaMate 8100 UV–vis Spectrophotometer.

### Mechanical Strength Tensile Test

Uniaxial tensile tests were conducted to investigate the mechanical strength of the prepared films by using an Instron machine (model 5969, equipped with a 500 N load cell) under a stretching speed of 20 mm min^−1^ at room temperature. For these tests, the films were cut into dumbbell‐shaped specimens, which followed the standard of JIS K 6251.

### Film Conductivity Measurement

The conductivity of the as‐prepared strain‐sensitive film, mounted with silver thread electrodes, was measured using electrochemical impedance spectroscopy (EIS) on an IviumStat electrochemical workstation. Measurements were conducted in a two‐electrode configuration with a 0 V bias, a sinusoidal excitation of 10 mV amplitude, and a frequency range from 0.1 Hz to 100 kHz. The resulting spectra were fitted in IviumSoft (R^2^ > 0.95) using the equivalent circuit shown in Figure S2 (Supporting Information). The film resistance, denoted as *R_film_*, was extracted from the fitting and used to determine conductivity. The cross‐sectional area (*A_c_*) and effective electrode separation (*L*) were measured, and the film conductivity was calculated as the following equation:

(1) $$\sigma_{film}=\frac{L}{R_{film}A_{c}}$$

### Dynamic Electrical Responses

Uniaxial tensile tests were conducted on an Instron machine (model 5969, equipped with a 500 N load cell) under a stretching speed of 20 mm min^−1^ at room temperature. The resistance signal of the strain sensors upon tensile and deformations was recorded by LabVIEW software through a digital multimeter (Model 2470 High Voltage SourceMeter Instrument). When assessing the sensing performance, the relative resistance change (Δ*R*/*R*_0_) was calculated using the equation below:

(2) $$\frac{\Delta R}{R_{0}}\left(\%\right)=\left(\frac{R_{1}}{R_{0}}-1\right)\times 100$$

where the *R*_0_ is the resistance of the original film without any elongation, and *R*_1_ is the resistance at each time point during the stretching and releasing process. The GF can be obtained as:

(3) $$\mathrm{GF}\;=\frac{\Delta R/R_{0}}{\varepsilon }$$

where *ε* is the strain at each time point. The dimensions of the film working section (discounting the length within the clamp on both sides) were 10 mm in length, 10 mm in width and ≈0.10 mm in thickness.

### Limit of Detection Study

Dynamic electrical responses of the PTGIL strain sensor under the strain of 0.2%–5% were tested. Error bars represent the standard deviation (SD) of the mean for *n* = 3 replicates. The limit of detection for PTGIL sensor is calculated below:

(4) $$Limitofdetection=\varepsilon +\left(3.3\times \sigma \right)$$

where *ε* is the smallest detectable strain, *σ* is the standard deviation error of signal of the smallest detectable strain. 3.3 is the uncertainty limit factor assuming a normal distribution. These findings show that the smallest detectable strain was 0.2%, then the limit of detection for the PTGIL sensor was 0.26% strain.

### Monitoring Human Motions

The sensor was designed to monitor human motions; therefore, sensor responses to the strain of finger bending, smiling, neck moving, wrist bending, elbow bending, and knee bending were investigated. The sensor was attached using commercial 3 M micropore surgical first aid medical tape and connected to conductive wires to monitor different human movements. The electrical connections made to the sensor was shown in Figure S8 (Supporting Information), and the resistance values of sensors during different motion detections were demonstrated in Figure S9 (Supporting Information). The resistance signals of the sensors were recorded using the digital multimeter as described above and processed in a similar manner. For these experiments, a single human volunteer was recruited following informed consent, in accordance with ethical approval granted by the University College London Research Ethics Committee (Approval Project No. 1135).

### Antioxidant Test

The antioxidant capability can be evaluated by investigating the efficiency of scavenging the stable DPPH free radical. The film was cut into small pieces, and 100 mg mL^−1^ solution was prepared by immersing 0.1 g of film in 10 mL of ethanol. Serial dilutions were applied to the 100 mg mL^−1^ stock solution to make 10, 5, 2.5, 1.25, 0.625, and 0.3125 mg mL^−1^ sample solutions. After that, 50 µL sample solution was added to each well of the 96‐well plate, and then 50 µL of the prepared 100 µM DPPH solution. After incubating (in a dark place) for 30 min, the absorbance was measured at 517 nm by a microplate reader (TECAN, 200 Pro, Switzerland). The percentage of DPPH scavenging was obtained from the equation below:

(5) $$\mathrm{DPPH}\;\mathrm{Scavenging}\;\left(\%\right)=\left(1-\frac{A_{B}}{A_{T}}\right)\;\times 100$$

where *A*_B_, *A*_T_ are the absorption of the blank (i.e., mixture of DPPH and ethanol) and the absorption of the test film solution (i.e., mixture of DPPH, ethanol, and sample), respectively.

### Self‐Healing Tests

The self‐healing PTGIL film was assessed after cutting a test film (20 mm (length) × 10 mm (width) × 0.10 mm (thickness)) into two pieces and making them moist. A 3D microscope (Keyence, VHX‐7000) was used to observe film self‐healing at ×700 magnification. Mechanical self‐healing efficiency was assessed by testing following the JIS K 6251 standard described above. On the other hand, the electrical recovery of PTGIL after self‐healing was evaluated through resistance measurements described above. The electrical self‐healing efficiency was calculated as the ratio of the average relative resistance change Δ*R*/*R*_0_ at 30% strain of the healed sensor to that of the original sensor. To further investigate the dynamically reversible interactions mechanisms of the PTGIL sensor, FTIR measurements were carried out at different time points (1, 10, 20, and 30 min after water induction) and compared with the spectra of the original film. To minimize spectral shifts caused by water evaporation in ambient environment and to eliminate additional peaks from residual water, the samples were placed in airtight boxes (Figure S12, Supporting Information), and excess water on the film surface was gently removed with tissue paper prior to measurement.

### Biocompatibility Cells Experiment

The cytotoxicity properties of the fabricated sensors were evaluated on the HDF cell line by LIVE/DEAD Viability/Cytotoxicity and AlamarBlue Cell Viability assays. The HDF cell line was used as it is associated with skin tissue repairing and wound healing. The cells were cultured in DMEM medium (supplemented with 10 vol.% FBS, 2 mM GlutaMAX and 1 vol.% antimycotic antibiotic under standard cell culture conditions of 37 °C and 5% CO_2_. HDF cells were cultured for 24 h before stimulation. LIVE/DEAD cell viability assay kit (Thermofisher, L3224) was used to evaluate the cell viability for the PTGIL sensor on the HDF cell line. Before seeding cells, a piece of strain sensor (the working dimension of the strain sensor is 10 mm in length, 10 mm in width, 0.10 mm in thickness, and the weight is 0.02 g) was sprayed with 70 vol.% Industrial Methylated Spirits (IMS) solution and exposed under UV light overnight, then they were immersed separately into 4 mL DMEM at 37 °C to get samples extracts. 0.22 µm filters were used to collect extracts. Next, 1 mL of each sample extract was added to 500 µL of DMEM media containing 10^4^ cells in each well of a 24‐well plate. Cells in the control groups were treated with 1.5 mL of DMEM. The LIVE/DEAD assay stock solution was prepared by mixing 5 µL of calcein AM (Component A) and 20 µL of ethidium homodimer‐1 (Component B) with 10 mL PBS solution. After 24 h incubation, the medium was removed and 200 µL LIVE/DEAD assay stock solution was added to each well and then incubated for 30 mins for staining. An EVOS M5000 microscope (Thermofisher Scientific) was used to observe fluorescence images. Each measurement was performed in triplicate. AlamarBlue Cell Viability assay was used to further test the cytotoxicity of PTGIL films. The procedures and concentrations of seeding cells are the same as the fluorescence test above. After 24, 48, and 72 h of incubation, 1.5 mL of 10 vol.% Alamarblue Cell Viability Reagent in complete DMEM was then added to each well (24‐well plate). All well plates were incubated at 37 °C with 5% CO_2_ for 3 h. Then, a microplate plate reader (TECAN, 200 Pro, Switzerland) was used to measure the fluorescence at 560/590 nm (Excitation/Emission). The fluorescence intensity = the fluorescence of the sample's well – the fluorescence of the reference well. All measurements were performed in triplicate.

### Antibacterial Test

*E. coli* (ATCC 25 922) and *S. aureus* (ATCC 43 300) were selected as representative bacteria of Gram‐negative bacteria and Gram‐positive bacteria for assessing antibacterial properties of PTGIL samples. Before the experiment, PBS solution (PBS tablets (Thermofisher)), broth solution (BD Difco Dehydrated Culture Media: Columbia Broth (Fisher Scientific)) and agar plate (Plate Count Agar (NutriSelect Plus, Sigma)) were prepared. To revive and prepare inoculum, a loopful of culture was added to 15 mL broth for 9.5 h at 37 °C at 220 rpm after completely thawing from −80 °C. Followed by subculturing and incubating for another 15 h at 37 °C at 220 rpm until the early stationary phase of growth (equivalent to 1 × 10^9^ CFU mL^−1^) was reached. Then, 0.02 g PTGIL film was immersed in 1 mL 1 × 10^9^ CFU mL^−1^ of bacteria suspension and incubated for up to 24 h as the treated group. 1 mL 1 × 10^9^ CFU mL^−1^ of bacteria suspension was incubated as the control group. The CFU method was then chosen to analyze the antibacterial property of the PTGIL film. The bacterial solutions were spread on LB Agar plates after serial dilution. Each LB Agar plate was incubated at 37 °C for 24 h for counting colonies. All experiments were performed in triplicate.

### Statistical Analysis

All data are presented as the mean ± standard deviation (SD) error of the mean (SEM) from a minimum of *n* = 3 experiments. Error bars corresponding to the cell viabilities assessed through of AlamarBlue assay represent the SD of the mean for *n* = 3 replicates. Statistical analysis for cell viability was carried out in OriginPro software v2021b using two‐way Analysis of Variance (ANOVA) coupled with Tukey's means comparison test to determine the level of statistical significance (i.e., *p* < 0.05).

## Conflict of Interest

The authors declare no conflict of interest.

## Supporting information



Supporting Information

Supplemental Movie 1

## Data Availability

The data that support the findings of this study are available from the corresponding author upon reasonable request.
