# Supplementary material for: Transparent Multifunctional Wearable Strain Sensor With Self‐Healing and Antibacterial Capabilities for Human Motion Detection
Source: Adv Healthc Mater. 2025 Oct 14;15(5):e03689. doi: 10.1002/adhm.202503689 (PMC12864581; doi:10.1002/adhm.202503689)
Supplement: Supplementary file 1 — Supporting Information [file ADHM-15-0-s002.docx]

Transparent Multifunctional Wearable Strain Sensor with Self-Healing and Antibacterial Capabilities for Human Motion Detection

*Wenqing Chen^1,2^, Wei Huang^1,2^, Rohit Gupta^1,2^, Abbas Heydari^1,2^, Biswajoy Bagchi^1,2^, Lulu Xu^1,2^, Yang Xue^1,2^, Eirini Velliou^3^, Manish K Tiwari^1,2,4 *^*

*^1^Nanoengineered Systems Laboratory, UCL Mechanical Engineering, University College London, London, WC1E 7JE, UK*

*^2^UCL Hawkes Institute, University College London, London, W1W 7TS, UK*

*^3^Centre for 3D Models of Health and Disease, Division of Surgery and Interventional Science, University College London, London W1W 7TY, UK*

*^4^Manufacturing Futures Laboratory, University College London, London, E20 2AE, UK*

** Corresponding author, e-mail address: m.tiwari@ucl.ac.uk*

**This file includes**:

**Figure S1**: Schematic illustration of the fabrication process of PTGIL films.

**Figure S2**: The equivalent circuit for conductivities measurement of PTGIL films.

**Figure S3**: Cyclic loading-unloading curves of the PTGIL film under strains of (a) 30% and (b) 100% for 15 cycles.

**Figure S4**: Limit of detection study based on resistive responses of the PTGIL strain sensor under different strain within 0.2-5% range. Error bars represent the standard deviation (SD) of the mean for n = 3 replicates.

**Figure S5**: The Δ*R*/*R*_0_ of the PTGIL sensor upon responding to the stepwise strain (0%–30%) at the speed of 20 mm/min used Day 2 sensors tested at (a) room temperature, (b) 0 ^o^C, (c) -10 ^o^C, and (d) -20 ^o^C.

**Figure S6**: The Δ*R*/*R*_0_ of the PTGIL sensor upon responding to the stepwise strain (0%–30%) at the speed of 20 mm/min used sensors made after 60 days tested at -20 ^o^C.

**Figure S7**: DPPH scavenging percentage by PTGIL film with different concentrations.

**Figure S8**: Sensor connection during motion detection. (a) wearing on the finger, (b) wearing on the waist.

**Figure S9**: Resistance values of the sensor when monitoring human motions. (a) smiling, (b) finger bending, (c) nodding, (d) wrist bending, (e) knee bending, and (f) elbow bending.

**Figure S10**: (a) FTIR spectra of original PTGIL sensor, water induced PTGIL sensor (1 min), water induced (10 mins) PTGIL sensor, water induced (20 mins) PTGIL sensor, water induced (30 mins) PTGIL sensor. (b) Zoom-in of the FTIR spectra from 3000 to 3700 cm^-1^.

**Figure S11**: Stretching, bending and twisting of healed PTGIL films.

**Figure S12**: Airtight box used for preventing water evaporation during self-healing FTIR tests.

**Table S1**: The experimental ingredients and nomenclatures of the as-prepared films.

**Table S2**: Comparison of performance parameters of different stretchable strain sensors.

**Movie S1**: Motion detection on finger bending.


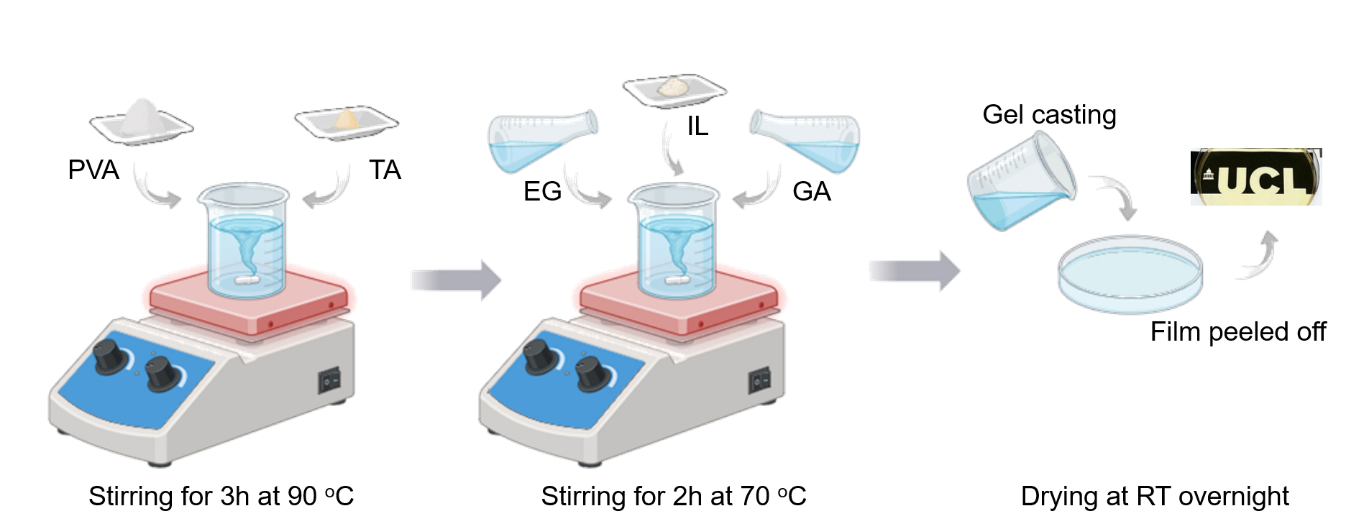


Figure S1. Schematic illustration of the fabrication process of PTGIL films.

To start with the sensor fabrication, an aqueous solutions were prepared by dissolving PVA in distilled water overnight with a concentration of 10 wt.%. Next, 1 wt.% tannic acid was added to the PVA solution and stirred at 90 ^○^C for 3 h to obtain a homogenous solution. Then, an appropriate amount of ethylene glycol, choline acetate and cross-linker glutaraldehyde were added to the PVA-TA solution and continually stirred at 70 ^○^C for 2 h. Subsequently, the mixture was cast in a glass mould and kept at room temperature overnight.


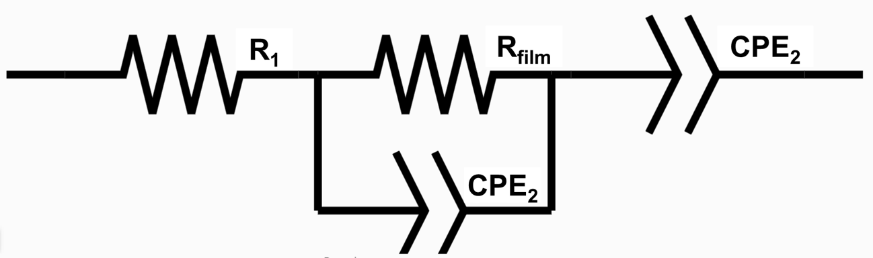


Figure S2. The equivalent circuit for conductivities measurement of PTGIL films.

In this circuit, *R_film_* is the primary quantity of interest used to determine the conductivity. The constant phase elements (CPEs) represent non-ideal interfacial capacitance (or charge storage ability).


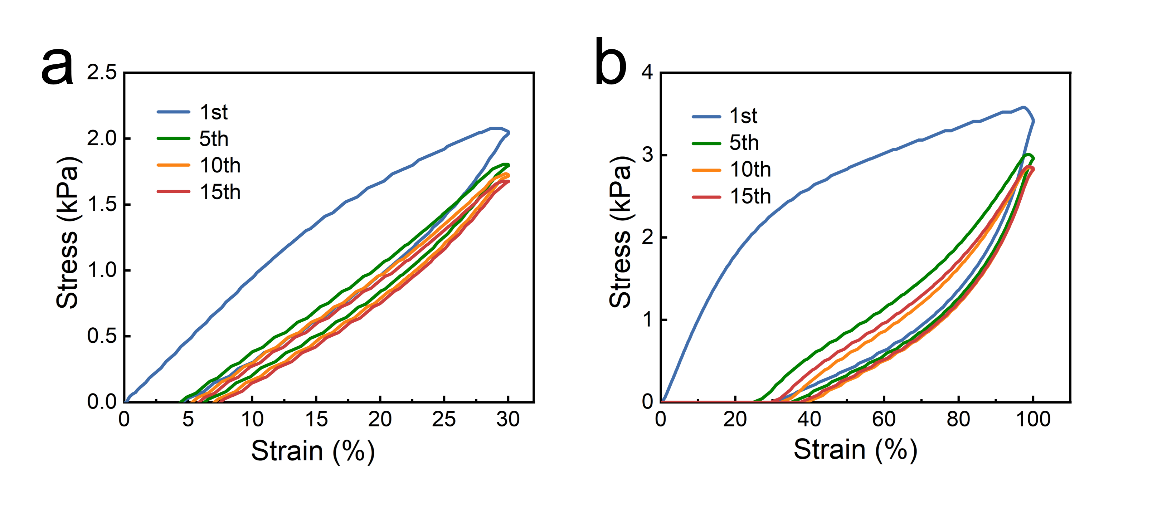


Figure S3. Cyclic loading-unloading curves of the PTGIL film under strains of (a) 30% and (b) 100% for 15 cycles.


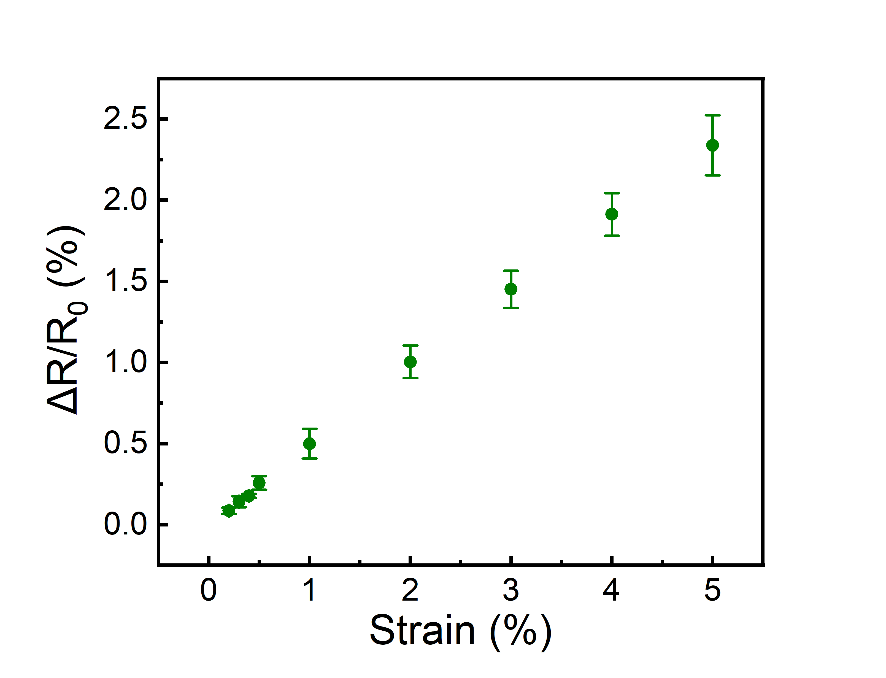


Figure S4. Limit of detection study based on resistive responses of the PTGIL strain sensor under different strain within 0.2-5% range. Error bars represent the standard deviation (SD) of the mean for n = 3 replicates.


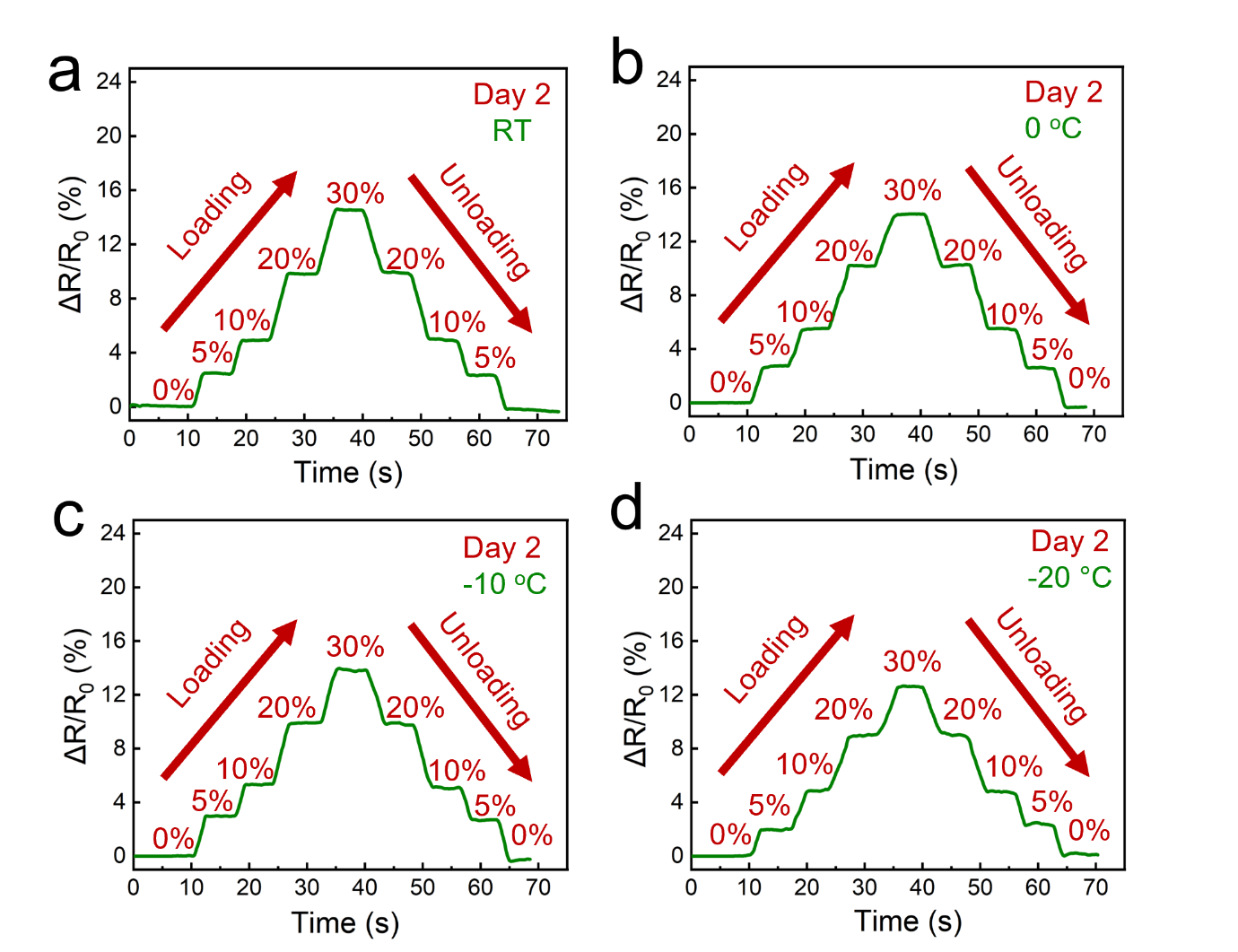


Figure S5. The Δ*R*/*R*_0_ of the PTGIL sensor upon responding to the stepwise strain (0%–30%) at the speed of 20 mm/min used Day 2 sensors tested at (a) room temperature, (b) 0 ^o^C, (c) -10 ^o^C, and (d) -20 ^o^C.


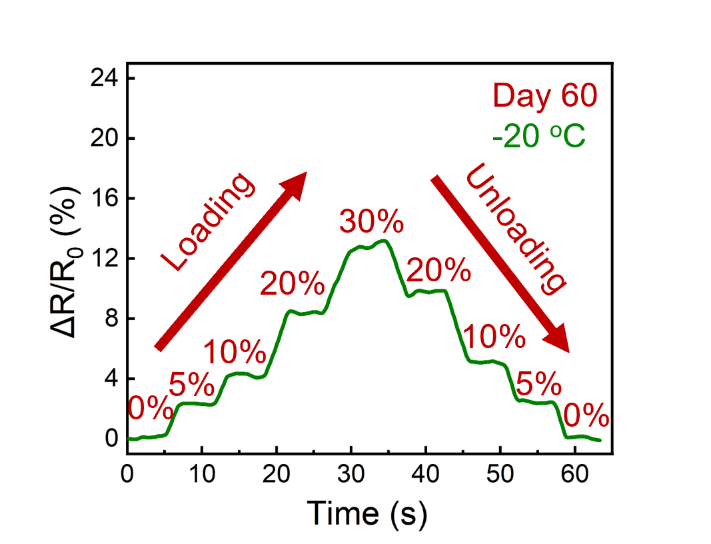


Figure S6. The Δ*R*/*R*_0_ of the PTGIL sensor upon responding to the stepwise strain (0%–30%) at the speed of 20 mm/min used sensors made after 60 days tested at -20 ^o^C.


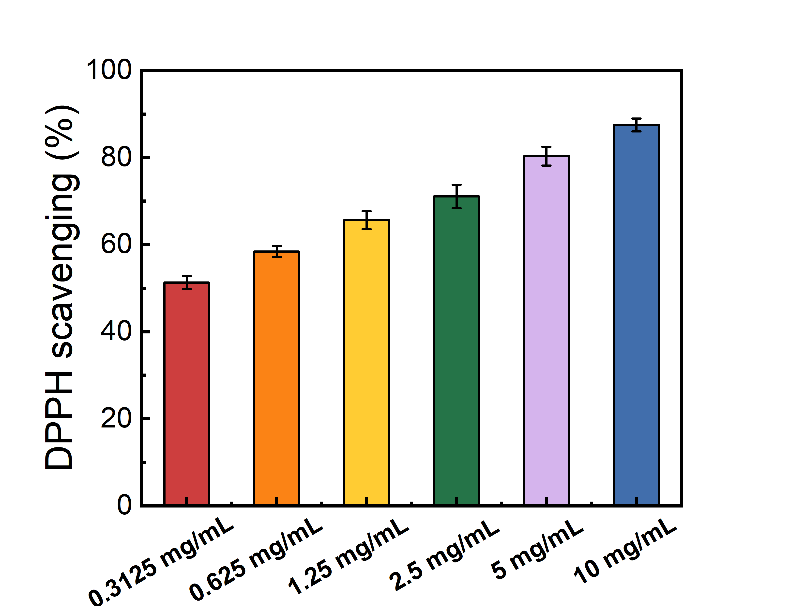


Figure S7. DPPH scavenging percentage by PTGIL film with different concentrations. Error bars represent the standard deviation (SD) of the mean for n = 3 replicates.

The antioxidant properties of PTGIL strain sensors were investigated by using the DPPH radical scavenging method. Films with a high percentage of DPPH radical scavenging activity were considered to have strong antioxidant property. With increasing concentrations of film extract, the antioxidant capacity of the films increased to nearly 89% after 30 min incubation with DPPH solution.


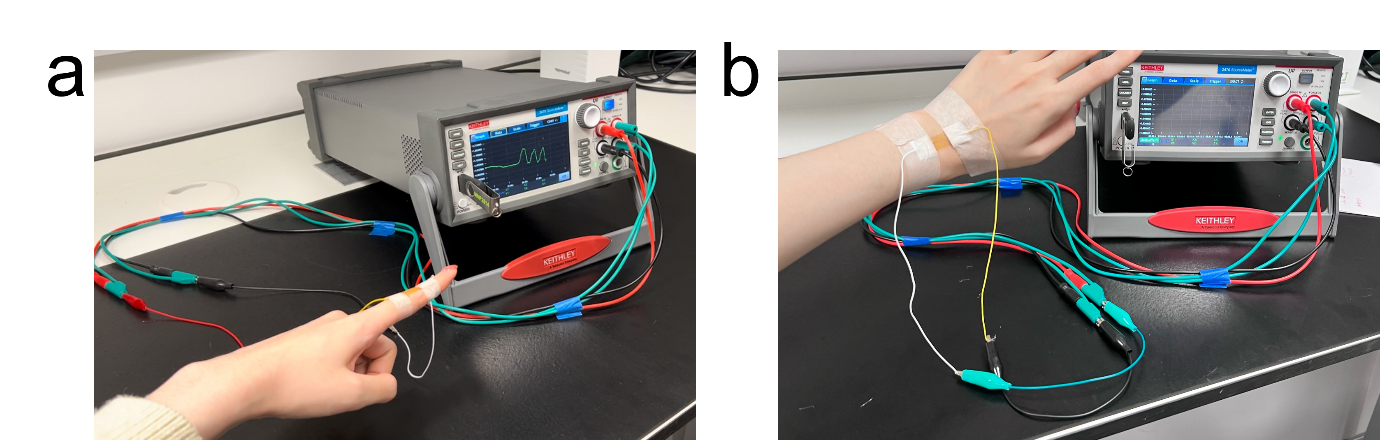


Figure S8. Sensor connection during motion detection. (a) wearing on the finger, (b) wearing on the waist.


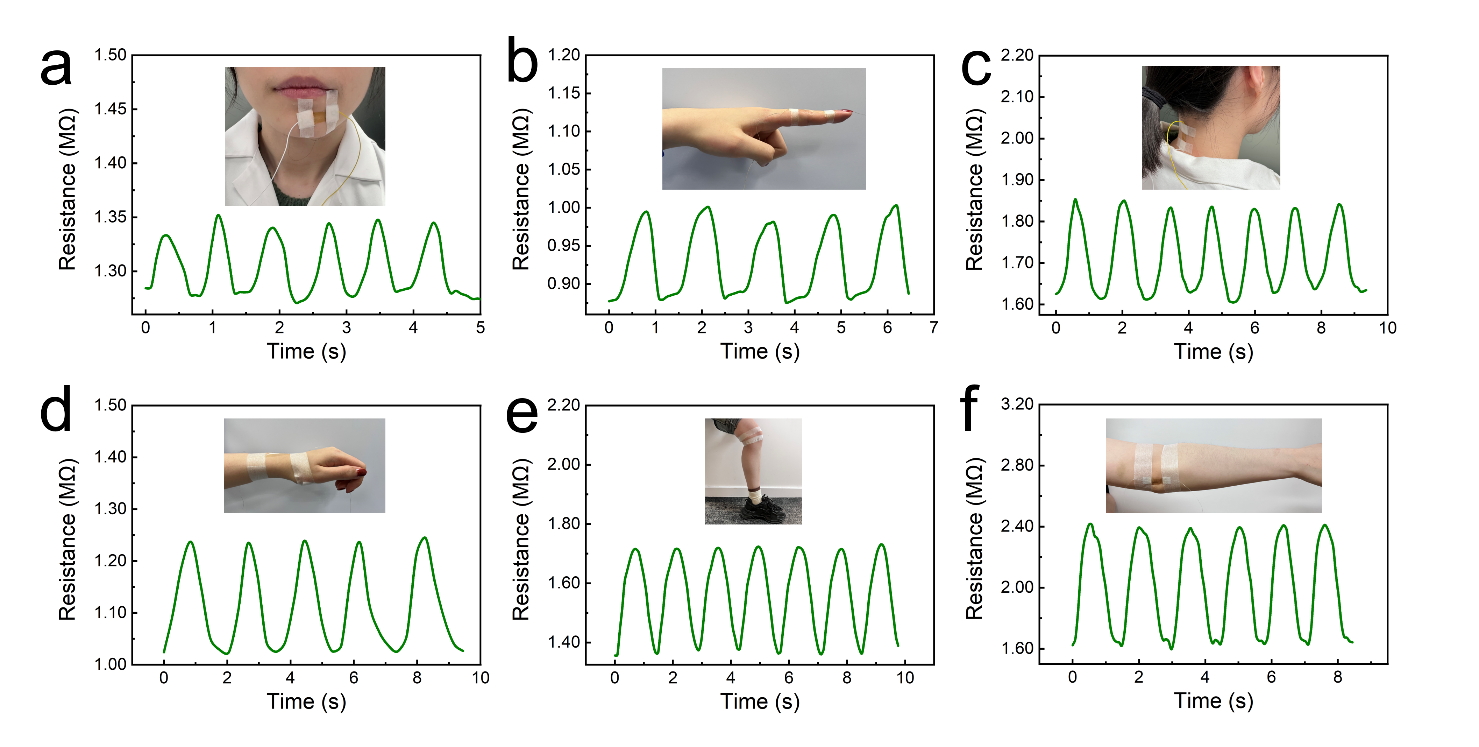


Figure S9. Resistance values of the sensor when monitoring human motions. (a) smiling, (b) finger bending, (c) nodding, (d) wrist bending, (e) knee bending, and (f) elbow bending.


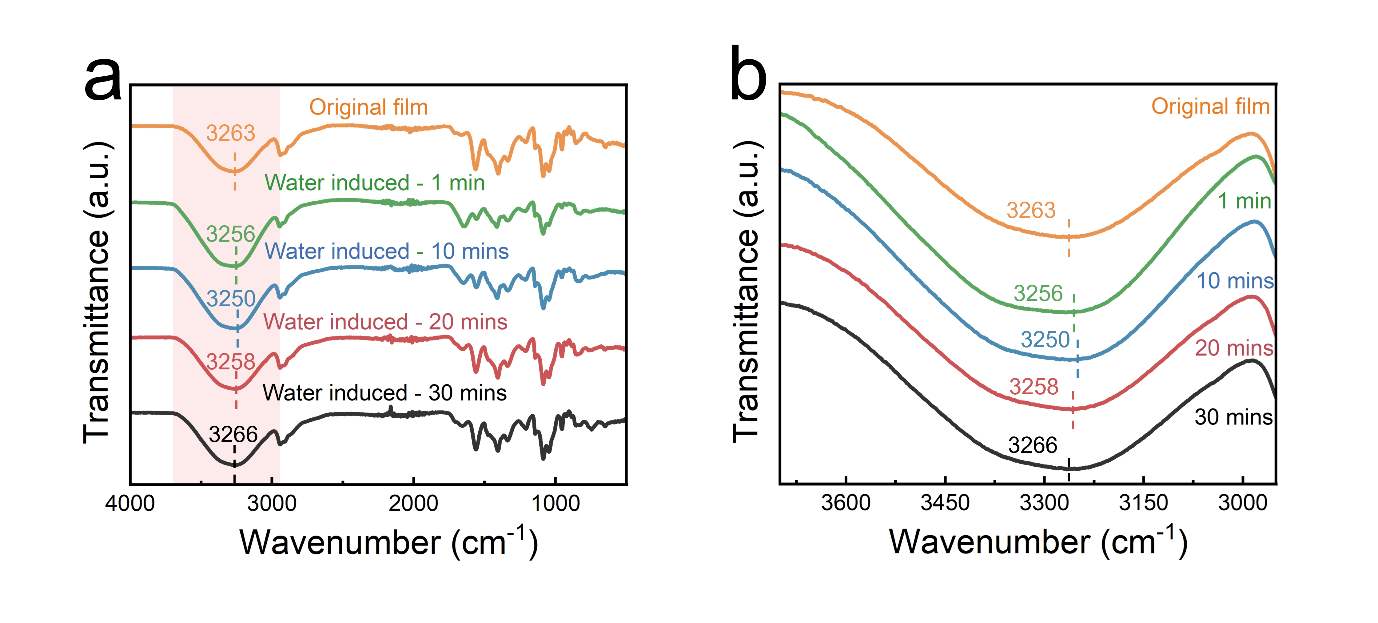


Figure S10. (a) FTIR spectra of original PTGIL sensor, water induced PTGIL sensor (1 min), water induced (10 mins) PTGIL sensor, water induced (20 mins) PTGIL sensor, water induced (30 mins) PTGIL sensor. (b) Zoom-in of the FTIR spectra from 3000 to 3700 cm^-1^.


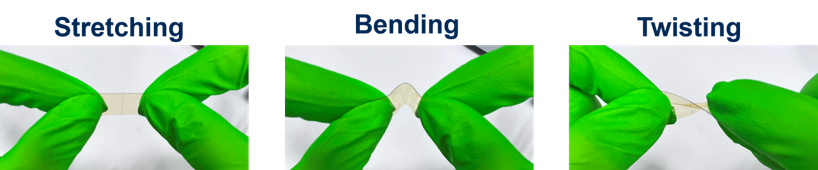


Figure S11. Stretching, bending and twisting of healed PTGIL films.


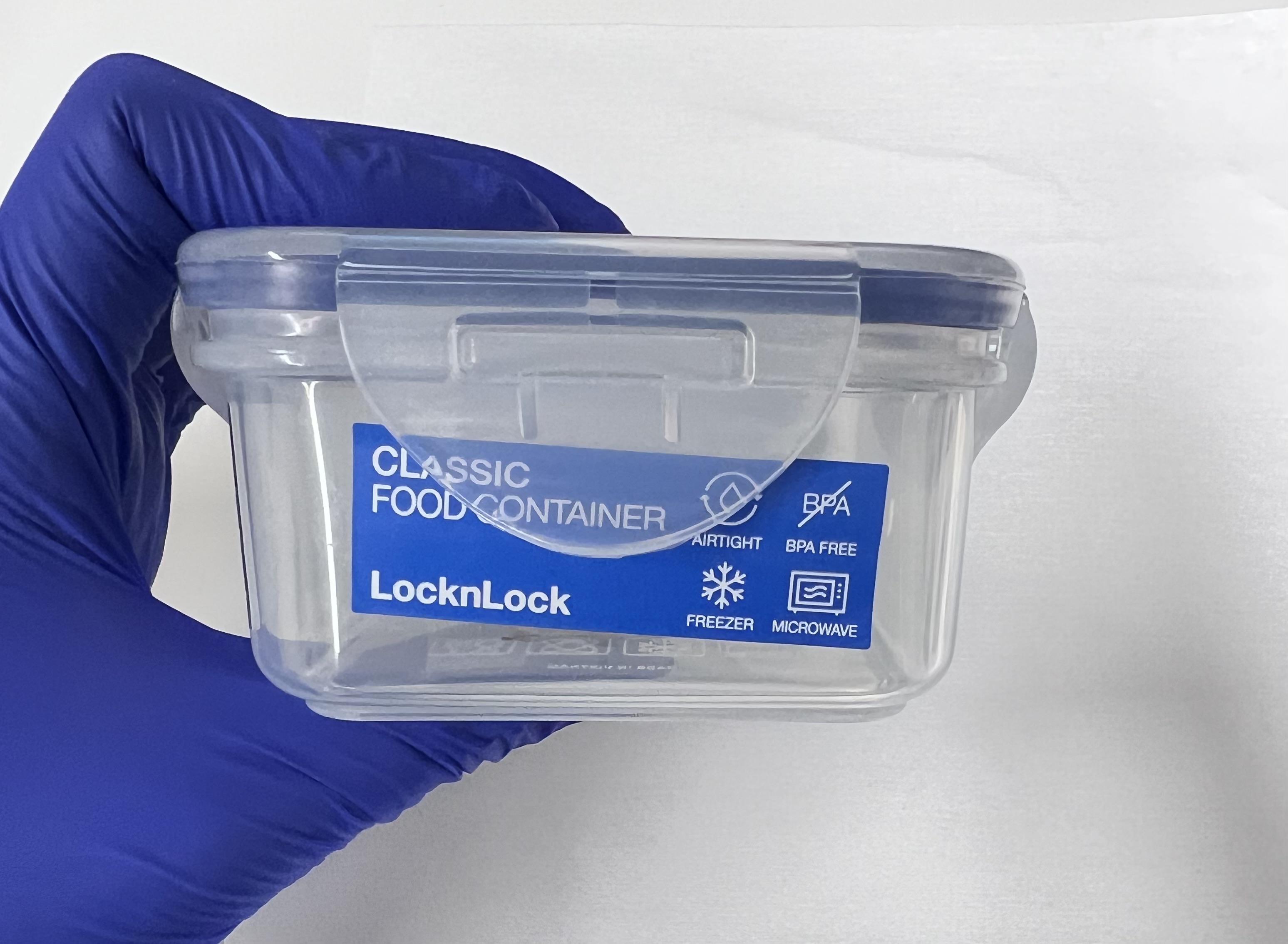


Figure S12. Airtight box used for preventing water evaporation during self-healing FTIR tests.

Table S1. The experimental ingredients and nomenclatures of the as-prepared films.

| Samples | PVA (g) | TA (g) | EG (g) | IL (g) | GA (g) |
| --- | --- | --- | --- | --- | --- |
| No IL | 1.5 | 0.15 | 0.3 | 0 | 0.15 |
| PTGIL_1_ | 1.5 | 0.15 | 0.3 | 0.3 | 0.15 |
| PTGIL_2_ | 1.5 | 0.15 | 0.3 | 0.6 | 0.15 |
| PTGIL_3_ | 1.5 | 0.15 | 0.3 | 0.9 | 0.15 |
| PTGIL_4_ | 1.5 | 0.15 | 0.3 | 1.2 | 0.15 |

Notes:

Polyvinyl alcohol (PVA), tannic acid (TA), ethylene glycol (EG), ionic liquid (IL), glutaraldehyde (GA)

**Supporting Information:**

Table S2. Comparison of performance parameters of different stretchable strain sensors.

| Materials | Gauge factor (GF) | Stretch-ability % | Transparent | Self-healing | Stability | Antiba-  cterial | Ref. |
| --- | --- | --- | --- | --- | --- | --- | --- |
| PTGIL (this work) | 0.5 (0-900%) | 900 | Yes | RT 20 min | 90 days | Yes | - |
| PVA/PLA/LiOH | 0.7 (0-1000 %) | ~ 1600 | - | - | - | - | ^[1]^ |
| PVASA-CS-rGO/PPy-CNT | 1.49 (0-176%) | 1491 | - | - | - | Yes | ^[2]^ |
| SSt/PAA/G | 2.12 (0-50%) | 120 | - | - | - | Yes | ^[3]^ |
| PVA/SA/PA/CaCl_2_/glycerol | 0.74 (0-50 %) | ~ 860 | Yes | - | 12 days | Yes | ^[4]^ |
| PVA/HPMC/PA/SBMA/ZnCl_2_ | 1.1 (0-600%) | ~ 750 | Yes | - | - | - | ^[5]^ |
| PAM/PVA/SC | 1.09 (0-200%) | 719 | - | - | - | - | ^[6]^ |
| PVA/CCN | 0.86 (0-100%) | 418 | - | - | - | - | ^[7]^ |
| PAM/Ca-Alg | 4.9 (0-4%) | 950 | - | - | 48 h | - | ^[8]^ |
| (NH_4_)_2_S_2_O_8_/allyl cellulose | 0.3 (0-90%) | 126 | Yes | - | - | - | ^[9]^ |
| SWCNTs/CB | 1.25 (0-120%) | 140 | - | - |  | - | ^[10]^ |
| PVA/Borax/PEDOT:PSS | 0.66 (0-23%) | 550 | - | RT 6h | - | - | ^[11]^ |
| PVA/PAMAA/Gly/Na^+^ | 1.8 (0-200%) | 1002 | - | 40ºC 4h | - | - | ^[12]^ |
| PAOAM-PDO | GF = 0.0222ε + 1.88 | 600 | Yes | RT 48h | 10 days | Yes | ^[13]^ |
| PVI-ChCl-Gly | 1.79 (0-150%) | 2310 | - | RT 12h | - | - | ^[14]^ |

Notes:

- If there are more than one GF over the whole working strain range (due to non-linearity), we only compare the GF for the low strain range.
- RT: room temperature. PVA/PLA/LiOH: Poly(vinyl alcohol)/poly(lithium acrylate)/LiOH. PVASA-CS-rGO/PPy-CNT: Poly(vinyl alcohol)-sodium alginate-chitosan-reduced graphene oxide/polypyrrole‐carbon nanotube. SSt/PAA/G: Sulfonated starch/poly(acrylic acid)/graphene. PVA/SA/PA/CaCl_2_/glycerol: Poly(vinyl alcohol)/alginate/phytic acid/CaCl_2_/glycerol. PVA/HPMC/PA/SBMA/ZnCl_2_: Poly(vinyl alcohol)/hydroxypropyl methyl cellulose/phytic acid/[2-(methylacryloxy) ethyl] dimethyl-(3-propyl sulfonate) ammonium hydroxide/ZnCl_2_. PAM/PVA/SC: Polyacrylamide/polyvinyl alcohol/sodium casein. PVA/CCN: Polyvinyl alcohol/Carboxymethyl chitosan/Citric acid monohydrate/Sodium Chloride. SWCNTs/CB: Single-walled carbon nanotube (SWCNTs)/ carbon black (CB). PVA/Borax/PEDOT:PSS: Poly(vinyl alcohol)/Borax/poly(3,4ethylenedioxythiophene):polystyrene sulfonate. PVA/PAMAA/Gly/Na^+^: Poly(vinyl alcohol)/poly(acrylic amide-acrylic acid)/glycerol/Na^+^. PAOAM-PDO: Organohydrogel/1,3-propanediol. PVI-ChCl-Gly: Polymerized 1-vinylimidazole/choline chloride/glycerol.

**REFERENCE**

[1] Z. Han, Y. Zhang, F. Yang, J. Chen, C. Sun, H. Xu, C. Liu, C. Shen, Covalent crosslinking modulated nanocrystallization of PVA/poly(lithium acrylate) hydrogel with Hofmeister effect for simultaneously excellent mechanical performance and ionic conductivity, *Chem. Eng. J.* **2025**, 507.

[2] W. Yu, J. Chen, Q. Gao, Y. Guo, S. Zhang, Y. Pan, B. Nie, X. Zhang, L. Jiang, J. Qiu, Z. Guo, R. Wei, Multifunctional PVA/SA-based hydrogels integrating high stretchability, conductivity, and antibacterial activity for human-machine interactive flexible sensors, *Chem. Eng. J.* **2025**, 519.

[3] J. Liu, S. Lv, Y. Mu, D. Wei, Y. Chen, T. He, J. She, L. Liu, Flexible and wearable strain sensor based on SSt/PAA/G composite hydrogel for human–machine interaction applications, *Chem. Eng. J.* **2025**, 522.

[4] H. Liu, S. Guan, P. Wang, X. Dong, Super Tough Anti-freezing and Antibacterial Hydrogel With Multi-crosslinked Network for Flexible Strain Sensor, *Small* **2025**, 21, e2407870.

[5] F. Wang, H. Zhang, C. Liu, W. Bao, Y. Hu, X. Maimaitiyiming, High toughness, high stability and low hysteresis PVA /HPMC/PA/SBMA/ZnCl(2) conductive hydrogels for wearable flexible electronics for multifunctional sensors and supercapacitors, *Carbohydr. Polym.* **2025**, 361, 123644.

[6] Z. Wu, L. Zhang, M. Wang, D. Zang, H. Long, L. Weng, N. Guo, J. Gao, Y. Liu, B. B. Xu, A wearable ionic hydrogel strain sensor with double cross-linked network for human–machine interface, *Adv. Compos. Hybrid Mater.* **2024**, 8, 17.

[7] Y. Yu, Z. Zhou, H. Ruan, Y. Li, High conductivity, low-hysteresis, flexible PVA hydrogel multi-functional sensors: Wireless wearable sensor for health monitoring, *Chem. Eng. J.* **2025**, 505, 158877.

[8] D. Yao, W. Wang, H. Wang, Y. Luo, H. Ding, Y. Gu, H. Wu, K. Tao, B. R. Yang, S. Pan, J. Fu, F. Huo, J. Wu, Ultrasensitive and Breathable Hydrogel Fiber‐Based Strain Sensors Enabled by Customized Crack Design for Wireless Sign Language Recognition, *Adv. Funct. Mater* **2024**, 35, 2416482.

[9] R. Tong, G. Chen, D. Pan, H. Qi, R. Li, J. Tian, F. Lu, M. He, Highly Stretchable and Compressible Cellulose Ionic Hydrogels for Flexible Strain Sensors, *Biomacromolecules* **2019**, 20, 2096.

[10] X. Guo, Y. Huang, Y. Zhao, L. Mao, L. Gao, W. Pan, Y. Zhang, P. Liu, Highly stretchable strain sensor based on SWCNTs/CB synergistic conductive network for wearable human-activity monitoring and recognition, *Smart Mater. Struct.* **2017**, 26, 095017.

[11] G. Ge, W. Yuan, W. Zhao, Y. Lu, Y. Zhang, W. Wang, P. Chen, W. Huang, W. Si, X. Dong, Highly stretchable and autonomously healable epidermal sensor based on multi-functional hydrogel frameworks, *J. Mater. Chem. A* **2019**, 7, 5949.

[12] J. Huang, S. Peng, J. Gu, G. Chen, J. Gao, J. Zhang, L. Hou, X. Yang, X. Jiang, L. Guan, Self-powered integrated system of a strain sensor and flexible all-solid-state supercapacitor by using a high performance ionic organohydrogel, *Mater. Horiz.* **2020**, 7, 2085.

[13] L. Zhao, Q. Ling, X. Fan, H. Gu, Self-Healable, Adhesive, Anti-Drying, Freezing-Tolerant, and Transparent Conductive Organohydrogel as Flexible Strain Sensor, Triboelectric Nanogenerator, and Skin Barrier, *ACS Appl Mater Interfaces* **2023**, 15, 40975.

[14] K. Fan, W. Wei, Z. Zhang, B. Liu, W. Feng, Y. Ma, X. Zhang, Highly stretchable, self-healing, and adhesive polymeric eutectogel enabled by hydrogen-bond networks for wearable strain sensor, *Chem. Eng. J.* **2022**, 449.
